# X-ray free-electron laser studies reveal correlated motion during isopenicillin *N* synthase catalysis

**DOI:** 10.1126/sciadv.abh0250

**Published:** 2021-08-20

**Authors:** Patrick Rabe, Jos J. A. G. Kamps, Kyle D. Sutherlin, James D. S. Linyard, Pierre Aller, Cindy C. Pham, Hiroki Makita, Ian Clifton, Michael A. McDonough, Thomas M. Leissing, Denis Shutin, Pauline A. Lang, Agata Butryn, Jürgen Brem, Sheraz Gul, Franklin D. Fuller, In-Sik Kim, Mun Hon Cheah, Thomas Fransson, Asmit Bhowmick, Iris D. Young, Lee O’Riordan, Aaron S. Brewster, Ilaria Pettinati, Margaret Doyle, Yasumasa Joti, Shigeki Owada, Kensuke Tono, Alexander Batyuk, Mark S. Hunter, Roberto Alonso-Mori, Uwe Bergmann, Robin L. Owen, Nicholas K. Sauter, Timothy D. W. Claridge, Carol V. Robinson, Vittal K. Yachandra, Junko Yano, Jan F. Kern, Allen M. Orville, Christopher J. Schofield

**Affiliations:** 1 Chemistry Research Laboratory, Department of Chemistry and the Ineos Oxford Institute for Antimicrobial Research, University of Oxford, 12 Mansfield Road, Oxford OX1 3TA, UK.; 2 Diamond Light Source, Diamond House, Harwell Science and Innovation Campus, Didcot OX11 0DE, UK.; 3 Research Complex at Harwell, Rutherford Appleton Laboratory, Didcot, Oxfordshire OX11 0FA, UK.; 4 Molecular Biophysics and Integrated Bioimaging Division, Lawrence Berkeley National Laboratory, 1 Cyclotron Road, Berkeley, CA 94720, USA.; 5 Linac Coherent Light Source, SLAC National Accelerator Laboratory, Menlo Park, CA 94025, USA.; 6 Department of Chemistry - Ångström, Molecular Biomimetics, Uppsala University, SE 751 20 Uppsala, Sweden.; 7 Interdisciplinary Center for Scientific Computing, University of Heidelberg, 69120 Heidelberg, Germany.; 8 Department of Bioengineering and Therapeutic Sciences, University of California, San Francisco, 600 16th Street, San Francisco, CA 94158, USA.; 9 Japan Synchrotron Radiation Research Institute, 1-1-1 Kouto, Sayo-cho, Sayo-gun, Hyogo 679-5198, Japan.; 10 RIKEN SPring-8 Center, 1-1-1 Kouto, Sayo-cho, Sayo-gun, Hyogo 679-5148, Japan.; 11 Stanford PULSE Institute, SLAC National Accelerator Laboratory, Menlo Park, CA 94025, USA.; 12 Department of Physics, University of Wisconsin–Madison, 1150 University Avenue, Madison, WI 53706, USA.

## Abstract

Isopenicillin *N* synthase (IPNS) catalyzes the unique reaction of l-δ-(α-aminoadipoyl)-l-cysteinyl-d-valine (ACV) with dioxygen giving isopenicillin *N* (IPN), the precursor of all natural penicillins and cephalosporins. X-ray free-electron laser studies including time-resolved crystallography and emission spectroscopy reveal how reaction of IPNS:Fe(II):ACV with dioxygen to yield an Fe(III) superoxide causes differences in active site volume and unexpected conformational changes that propagate to structurally remote regions. Combined with solution studies, the results reveal the importance of protein dynamics in regulating intermediate conformations during conversion of ACV to IPN. The results have implications for catalysis by multiple IPNS-related oxygenases, including those involved in the human hypoxic response, and highlight the power of serial femtosecond crystallography to provide insight into long-range enzyme dynamics during reactions presently impossible for nonprotein catalysts.

## Teaser

Complementary XFEL methods reveal O_2_ reaction intermediates and global protein dynamics during Isopenicillin *N* synthase catalysis.

## INTRODUCTION

Following pioneering studies demonstrating l-δ-(α-aminoadipoyl)-l-cysteinyl-d-valine (ACV) is the precursor of all natural penicillins, isopenicillin *N* synthase (IPNS) was shown to catalyze formation of both penicillin β-lactam and thiazolidine rings, in an iron- and dioxygen-dependent reaction without synthetic precedent (*1*–*3*). IPNS is a member of the Fe(II) and 2-oxoglutarate (2OG) oxygenase structural superfamily (*4*); such enzymes are widespread in nature and have important roles, including in collagen biosynthesis, lipid metabolism, nucleic acid repair, and signaling (*5*, *6*). 2OG oxygenases play key roles in the human hypoxic response via hydroxylation of the hypoxia inducible factors in a manner regulated by dioxygen availability (*7*, *8*). Understanding how dioxygen interacts with the 2OG oxygenase superfamily is of interest from catalytic and physiological perspectives.

**Fig. 1:**
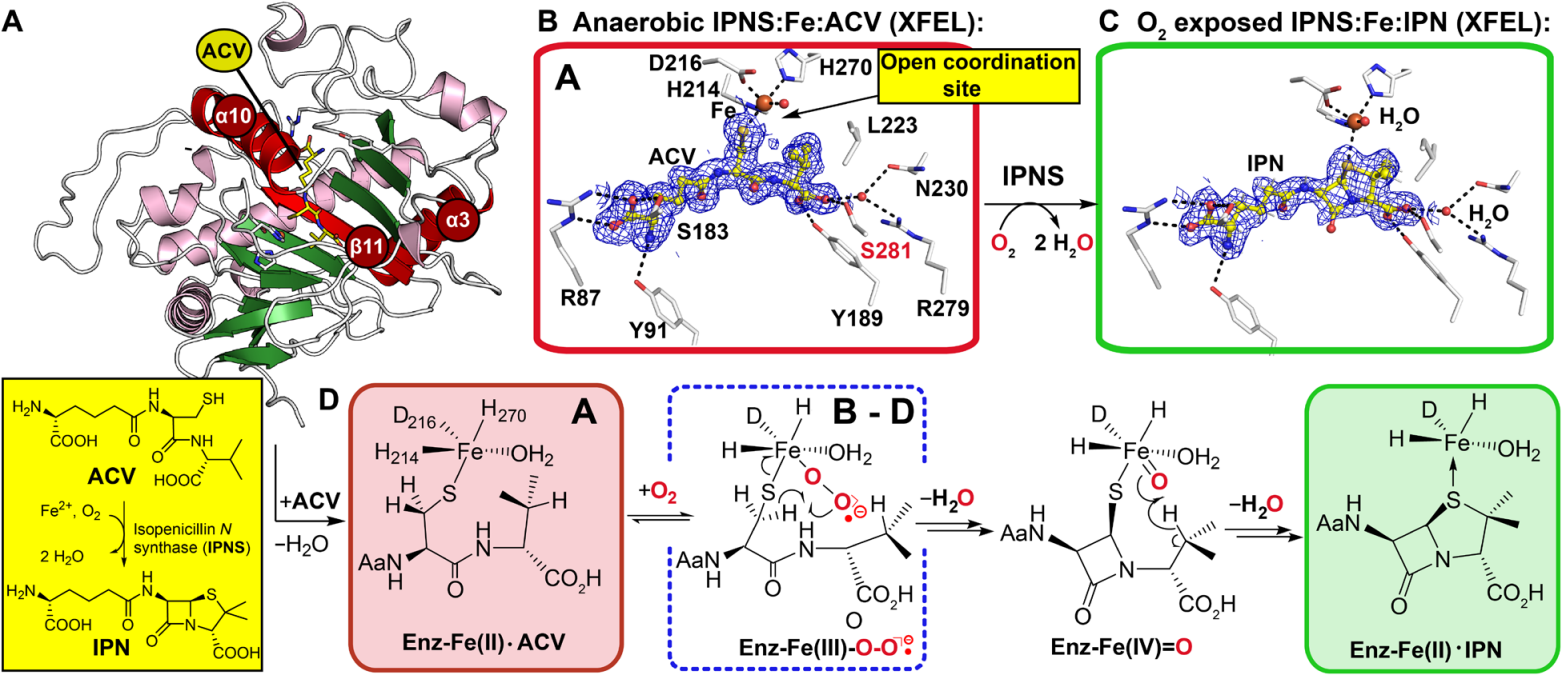
IPNS catalysis is amenable to tr-SFX analysis. (**A**) Protein fold of anaerobic IPNS:Fe(II):ACV (conformation A, PDB: 1BLZ) (*26*); Fe, orange sphere; ACV, yellow; α-helices, purple; β strands, green; except elements involved in ACV binding/flexible regions due to O_2_ binding; red. (**B**) Composite SFX 2mF_o_-DF_c_ omit electron density map for anaerobic IPNS:Fe:ACV (PDB: 6ZAE, 1.0 σ contour level, 1.40-Å resolution; fig. S2), showing key ACV interactions (R87, Y91, S183, Y189, L223, N230, R279, and S281) highlighting S281 (red), which is important in dynamics. (**C**) Composite SFX 2mF_o_-DF_c_ omit map for IPNS:Fe:IPN obtained by ~30-min O_2_ exposure of IPNS:Fe:ACV microcrystals (PDB: 6ZAQ, 1.0 σ contour level, 1.60-Å resolution; fig. S2). (**D**) Key intermediates with the superoxide (ACV conformations B to D, further addressed in Fig. 3 due to ACV rearrangement after O_2_ binding) in a blue dashed box. Note: The exact localization of the electrons in the Fe superoxo complex is unknown. To reflect this, we have used the nomenclature Fe-O_2_^⸣●−^.

2OG oxygenases typically catalyze hydroxylation reactions that are coupled to 2OG oxidation giving succinate and CO_2_ (*4*, *5*). IPNS catalyzes a unique four-electron oxidation involving two challenging C─H bond cleavages during conversion of the inactive peptide ACV to the conformationally strained antibiotic isopenicillin *N* (IPN) (Fig. 1 and fig. S1). Substrate analog (*2*, *9*), kinetic (*10*), spectroscopic (*11*, *12*), and modeling (*10*, *11*, *13*–*17*) studies imply binding of dioxygen to the IPNS:Fe(II):ACV complex that yields an Fe-linked superoxide, which abstracts a C-3 hydrogen from the ACV cysteine giving a thioaldehyde, which undergoes 4-*exo*-tricyclization to produce a β-lactam linked via S to an Fe(IV)═O species. Thiazolidine formation occurs via valine hydrogen abstraction followed by reductive elimination giving the bicyclic IPN ring system (fig. S1) (*4*, *6*, *10*). There is a lack of knowledge of how the IPNS protein mediates the conformational changes required for penicillin formation, and structural knowledge of the intermediate formed by reaction of IPNS:Fe(II):ACV with dioxygen has been unavailable.

Redox labile intermediates are not amenable to synchrotron analysis because they react via x-ray–induced photoelectric effects (*18*). X-ray free-electron lasers (XFELs) provide intense femtosecond-long x-ray pulses enabling diffraction data from thousands of micron-size crystals, limiting radiation-induced chemistry (serial femtosecond crystallography, SFX) (*19*). To enable time-resolved (tr) SFX studies (*20*–*22*) on IPNS catalysis, we exposed anaerobic IPNS:Fe(II):ACV microcrystals to dioxygen using acoustic droplet ejection (ADE) tape drive methods (*23*) with simultaneous monitoring of the Fe oxidation state by x-ray emission spectroscopy (XES) (*24*, *25*). We subsequently used ^19^F NMR (nuclear magnetic resonance) to probe the solution relevance of the dynamics revealed by tr-SFX.

## RESULTS

To focus tr-SFX analyses on events associated with O_2_ reaction rather than ACV binding and/or IPN release, we co-crystallized the anaerobic IPNS:Fe(II):ACV complex under anaerobic conditions to produce a dense microcrystal slurry (~3 μm × 3 μm × 60 μm, needle morphology, *P*2_1_2_1_2_1_ space group). The microcrystals that diffract to high resolution (tables S1 and S2) are amenable to efficient reaction initiation by exposure to O_2_ and trap IPN at the active site due to lattice constraints (fig. S2), ensuring single turnover conditions.

The anaerobicity of our ADE tape drive sample delivery system and suitability of IPNS microcrystals for tr-SFX analysis was validated by determination of an SFX structure of anaerobic IPNS:Fe:ACV (Fig. 1 and fig. S2), which is similar to reported synchrotron structures under cryo- and room temperature conditions [C-α RMSDs: 0.338 Å/Protein Data Bank (PDB) 1BK0 (*26*) and 0.234 Å/6Y0O (*27*)]. The active site iron refines to full occupancy and exhibits square pyramidal coordination with ligation by His^214^, Asp^216^, His^270^, a water, the ACV thiolate, with an open coordination site trans to Asp^216^. ACV interactions relevant to the tr-SFX analyses include a salt bridge of its α-aminoadipoyl (Aad) carboxylate with Arg^87^ (which hydrogen bonds with Thr^331^ on the C-terminal helix α10), hydrogen bonds between Tyr^91^ and the Aad amino group, and of the ACV valine carboxylate with Tyr^189^ and Ser^281^ (fig. S1).

Exposure of room temperature IPNS:Fe:ACV microcrystals to air for ≥30 min resulted in high-resolution structures and IPN refined to full occupancy, with diffraction data collected at cryogenic or room temperatures, at both synchrotrons and XFELs, implying a single, O_2_-dependent ACV turnover (SFX rt.: PDB: 6ZAQ; MX cryo: PDB: 6ZAO; SSX fixed target: PDB: 6Y0P; Fig. 1C and fig. S2). In accord with prior work (*28*), exposure of large single IPNS:Fe(II):ACV crystals to O_2_ resulted in incomplete conversion to IPN (*26*), even with high pressure/prolonged reaction, implying more efficient O_2_ diffusion in microcrystals. For the IPN product complexes reported here, we refined a five-coordinate distorted square pyramidal Fe site, ligated by His^214^, Asp^216^, His^270^, a water, and the IPN sulfur (Fe-S: 2.98 Å), differing from the square pyramidal coordination in IPNS:Fe:ACV and modeling of the proposed IPN complex (*10*). In our IPN product complexes derived from microcrystals, the Fe refined to convergence with low occupancy (40 to 60%). However, solution studies using NMR and non-denaturing mass spectrometry (MS) provide no evidence for IPN-promoted Fe displacement (fig. S2). Thus, the apparent loss of Fe on formation of IPN in crystallo likely reflects tight IPN binding due to lattice packing interactions, which promote release of Fe rather than IPN.

We investigated the reaction of IPNS:Fe(II):ACV with O_2_ by exposing microdroplets of the anaerobic microcrystal slurry to a 100% O_2_ atmosphere, regulating the reaction time by changing the ADE tape drive speed (*23*). On the basis of the high ratios of indexed crystal lattices to images collected, multiple datasets (for 400-, 500-, 800-, 1600-, and 3000-ms O_2_ exposure times) were collected from the same microcrystal batch (table S1) enabling robust dataset comparison.

**Fig. 2:**
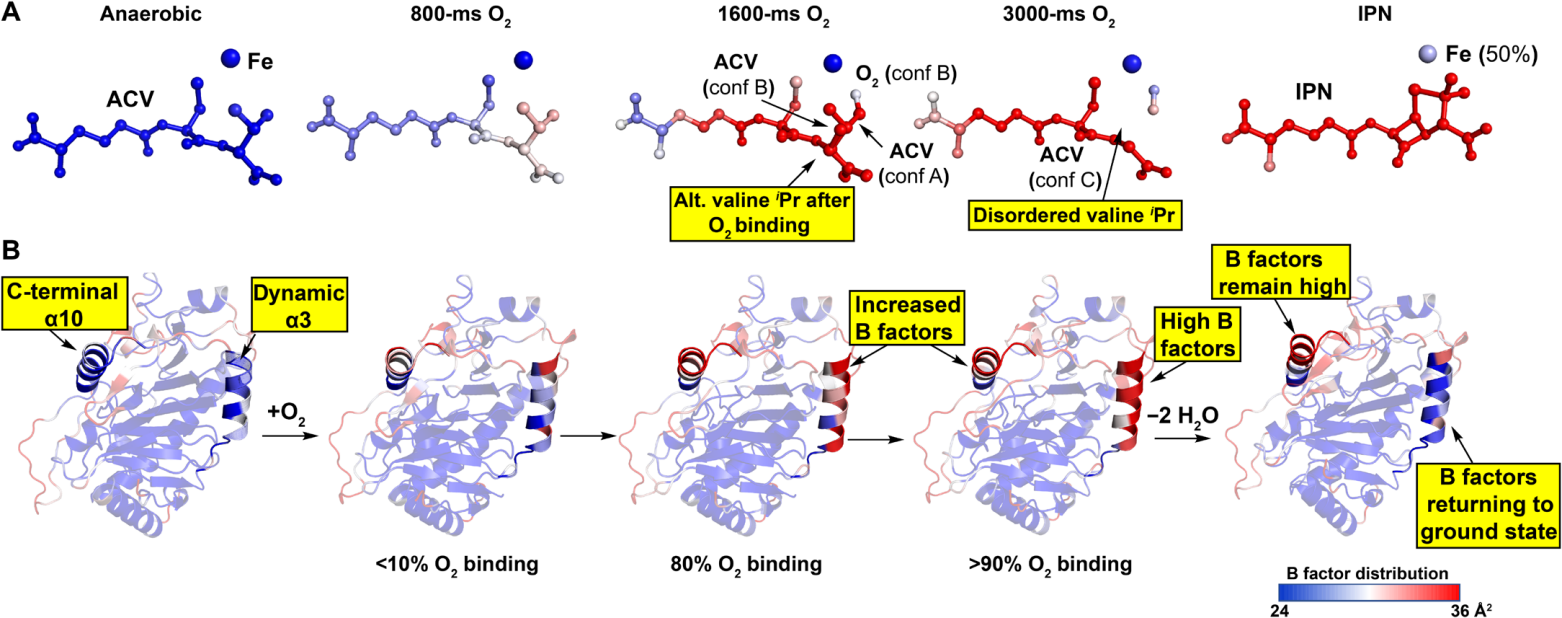
O_2_ binding to IPNS:Fe:ACV causes dynamics throughout the protein. Color gradient B-factor analysis of tr-SFX data from blue (24 Å^2^), to white, to red (36 Å^2^) for (**A**) Fe, O_2_, substrate/intermediate/product and (**B**) the protein including α3 (amino acids 47 to 64) and the C-terminal region (α10; amino acids 315 to 331). Note: (i) α3 B-factors rise on O_2_ exposure and decrease on IPN formation, (ii) ACV valine–derived atoms become disordered on O_2_ exposure but are ordered in the IPN complex, (iii) α10 is involved in ACV binding/IPN product release and remains disordered in IPNS:Fe:IPN complex since IPN is trapped due to lattice packing constraints.

Diffusion of O_2_ into microcrystals slightly alters the unit cell parameters, suggesting changes that alter fold and/or lattice packing. No clear structural differences at 400, 500, or 800 ms were apparent (fig. S3); however, analysis of the ACV B-factors at 500 ms and longer times reveals substantial increases (22 Å^2^/anaerobic to 36 Å^2^/1600 ms; Fig. 2 and fig. S3). The ACV valine B-factors increased first (500 ms), followed by those of the entire ACV (≥ 800 ms), including the Aad side chain. The latter was unexpected, because all ACV/IPN atoms are fully ordered in both, the anaerobic ACV substrate and IPN product complexes (Fig. 1 and figs. S2 and S3). These observations suggest that O_2_ binding induces motions beyond those directly involved in covalent reaction at the active site. We observed that addition of glycerol (to minimize dehydration) reduces the reaction rate, consistent with O_2_ diffusion being slowed by increased viscosity in the presence of glycerol (fig. S4) (*29*).

**Fig. 3:**
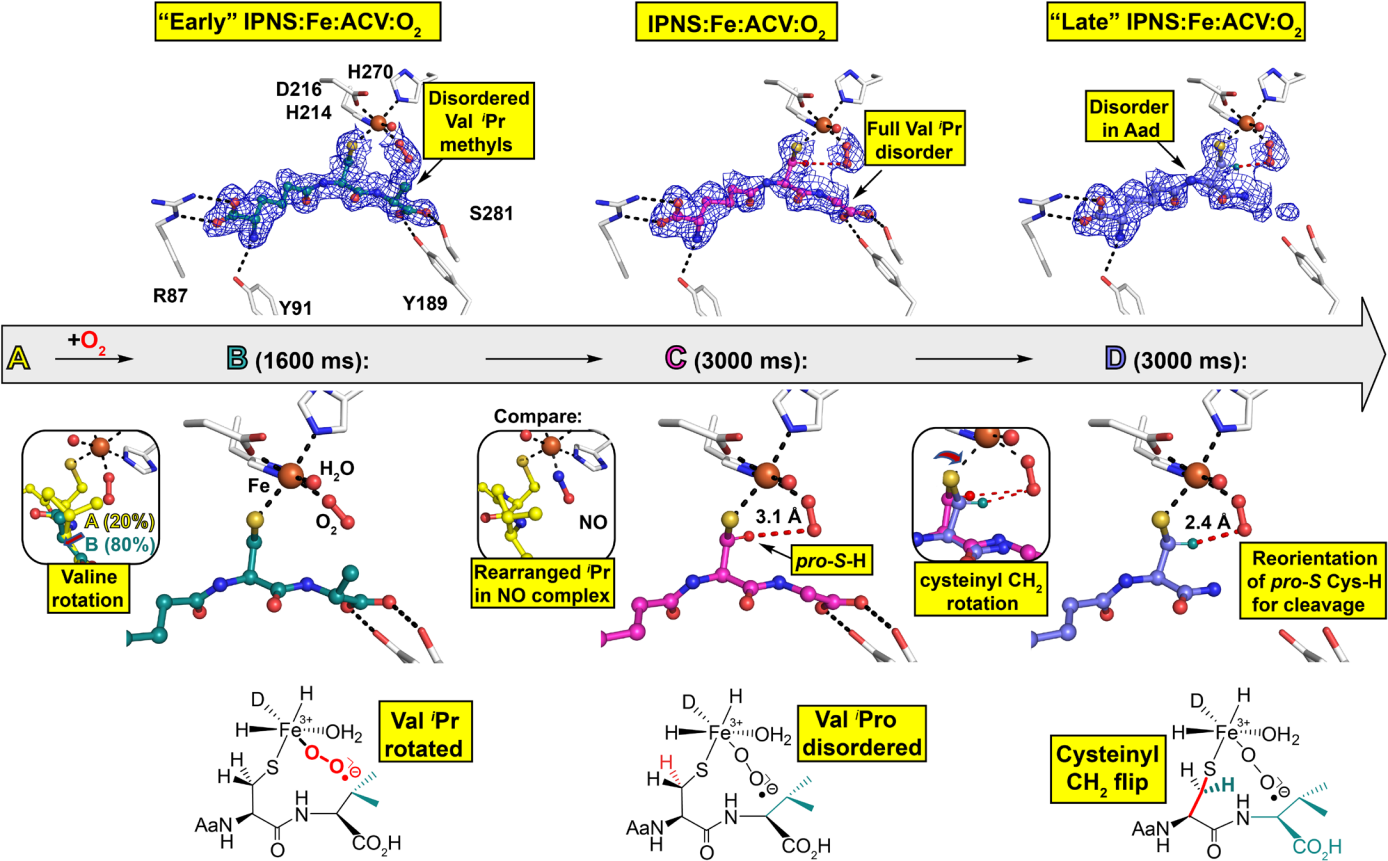
tr-SFX reveals mode of O_2_ binding to IPNS:Fe:ACV. ACV-derived conformations (**A** to **D**) were fit and refined to the 1600-ms (ACV conformation A and B) and 3000-ms (ACV conformation C and D) tr-SFX datasets (PDB: 6ZAI and 6ZAJ). 2mF_o_-DF_c_ omit maps (1.0 σ contour level, 1.53 and 1.55 Å resolution, respectively) are shown. Conf. A: The same as the anaerobic ACV complex (Fig. 1). Conf. B: The major 1600-ms IPNS:Fe:ACV + O_2_ conformation (B, 80%) refined with the ACV Val isopropyl methyls deleted (teal). Left inset: Overlay of IPNS:Fe:ACV (A, 20%, details in Fig. 1B) and IPNS:Fe:ACV:O_2_ (B, 80%) 1600-ms models. Conf. C: (3000 ms), where all Val isopropyl atoms are disordered. Central inset: The anaerobic IPNS:Fe:ACV:NO complex (PDB: 6ZAN, cryo MX, 1.39-Å resolution). Conf. D: New proposed conformation in the 3000-ms dataset with the Cys side-chain rotated. Right inset: Superimposition of confs. C and D, showing how the ACV cysteinyl methylene rotation positions the pro-*S* C_Cys,β_─H bond close to the distal O of the Fe-O_2_ for stereospecific C─H cleavage (fig. S6).

Positive electron density appeared at the Fe coordination site trans to Asp^216^ in the 1600- and 3000-ms SFX datasets (Fig. 3 and fig. S5). The 1600-ms dataset electron density was initially fit and refined with a coordinated water, but difference features remained and this model did not adequately reflect the scattering. The best refined fit to the 1600-ms dataset includes an O_2_ bound “end-on” to the Fe (figs. S5 and S6), consistent with modeling (fig. S7) (*11*, *16*).

In refinements for the 1600- and 3000-ms datasets, we omitted those ACV atoms with very weak or lacking 2mF_o_-DF_c_ density, which did not generate mF_o_-DF_c_ difference features when removed. These atoms are present but are disordered in intermediates rendering them invisible to x-ray diffraction (XRD), despite the use of 35-fs x-ray pulses. By contrast, before reaction initiation and following its completion, all nonhydrogen ACV or IPN atoms are visible. Thus, as O_2_-initiated reaction progresses, the ACV becomes dynamic, with the extent of disorder varying with the substrate region.

The results imply that O_2_ binding causes the ACV Val isopropyl group to rotate away from the Fe to avoid a steric clash; similar rotation is observed in synchrotron cryo-structures for the IPNS:Fe:ACV:NO complex, wherein NO is an O_2_ surrogate (Fig. 3 and fig. S7). By contrast with the IPNS:Fe:ACV:NO complex, in the 1600-ms dataset, electron density for the ACV Val is weak and the Val isopropyl methyls were deleted in the refinement models [ACV conformation (conf.) A, 20% occupancy (− O_2_) and conf. B, 80% occupancy (+O_2_); Fig. 3 and fig. S5].

Similar considerations apply to the 3000-ms dataset, where we fit and refined the ACV with the entire Val isopropyl deleted. In this dataset, we also observed decreased electron density, consistent with partial disorder, for both the valine carboxylate and Aad side chain (conf. C; Fig. 3 and fig. S5). In the 3000-ms dataset, an additional conformation (conf. D) was proposed in which the ACV cysteinyl methylene rotates such that the pro-*S* C_Cys,β_─H bond is close to the distal O of the Fe-O_2_ (2.4 Å compared to 3.1 Å in conf. C). This produces a complex aligned for stereospecific cysteinyl 3─H bond cleavage (*10*) leading to a thioaldehyde, which is preorganized for stereoelectronically favored β-lactam formation (Fig. 3 and figs. S5 and S6). Thus, the tr-SFX results reveal that, at this stage of the reaction, the ACV Cys/Val atoms involved in thioaldehyde and β-lactam formation appear to be more ordered than those Aad and Val atoms that are not directly involved in these steps of the reaction cycle.

**Fig. 4:**
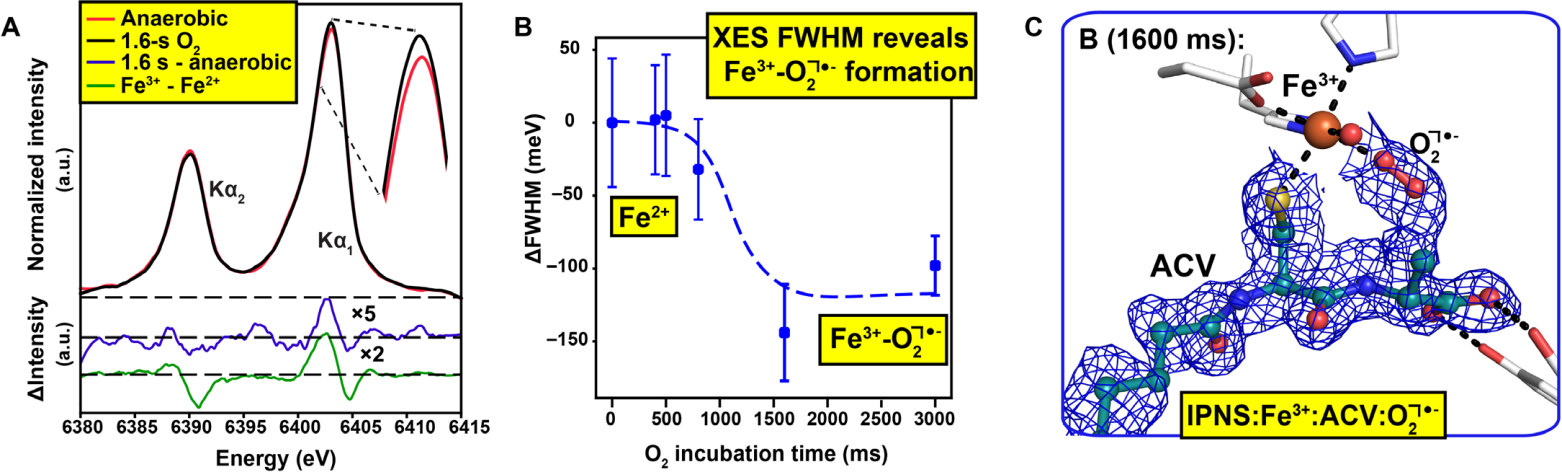
Tr-XES analysis reveals superoxide formation. (**A**) Fe Ka_1,2_ XES of anaerobic IPNS:Fe:ACV microcrystals (red), after 1600-ms O_2_ exposure (black); a difference spectrum between the two (blue, 5× enlarged) and a difference spectrum between 10 mM solutions of Fe(III)Cl_3_ and Fe(II)Cl_2_ (green, 2× enlarged). a.u., arbitrary units. (**B**) Full width at half maximum (FWHM) of the Fe Kα_1_ XES peak following O_2_ exposure. Early times (≤800 ms) show only small changes in the FWHM (+/− 0.03 eV); the 1600- and 3000-ms times show a shift by ~0.1 eV, indicating Fe oxidation. (**C**) 2mF_o_-DF_c_ map of the IPNS:Fe^3+^:ACV:O_2_^⸣●−^ complex (PDB: 6ZAI).

The tape drive setup enables simultaneous collection of tr-SFX and tr-XES data (*23*), allowing assignment of the Fe oxidation states (*30*). XES analysis of the anaerobic IPNS:Fe:ACV microcrystal complexes supports the Fe(II) oxidation state (Fig. 4A and 4B, and fig. S6). In the O_2_-bound datasets (1600 and 3000 ms and 500 ms without glycerol; figs. S4 and S6), changes are observed in the XES and difference spectra (Fig. 4A), especially in the full width at half maximum (FWHM) (*25*) of the Kα_1_ peak (Fig. 4B). These changes are similar in magnitude to those on going from Fe(II) to Fe(III) in Mn/Fe containing R2a ribonucleotide reductase (*23*), in the diiron center of methane monooxygenase (*31*), and in small-molecule Fe complexes (*32*), supporting formation of an IPNS:Fe(III):ACV:O_2_^−^ intermediate consistent with solution studies (*10*, *11*, *17*) and our tr-SFX data.

The tr-SFX datasets reveal clear changes of average B-factors in two structurally separate regions: (i) the C-terminal region including α10 [which is important for productive catalysis (*3*, *33*)] and the loop connecting α10 with the protein core and (ii) α3, for which no function is ascribed and which is located on the exterior of the double stranded b helix core (DSBH) fold (Fig. 2 and figs. S3 and S4). Two other exterior regions also refine with increased B-factors (amino acids 105 to 120, including α5) and an exterior loop (amino acids 194 to 206), although these do not exhibit changes correlating well with increasing O_2_ exposure time. The α10 B-factors increase with time and remain high in the IPNS:Fe:IPN complex. Those for α3 increase with time, but in the IPNS:Fe:IPN complex (Fig. 2 and fig. S3), they refine back to values close to those for anaerobic IPNS:Fe:ACV. Comparison of the *F*_obs_ − *F*_obs_ isomorphous difference maps for the 400- and 3000-ms datasets reveals clear evidence for dynamic changes in the α3/β11 conformations, consistent with the calculated mF_o_-DF_c_ maps (fig. S3).

**Fig. 5:**
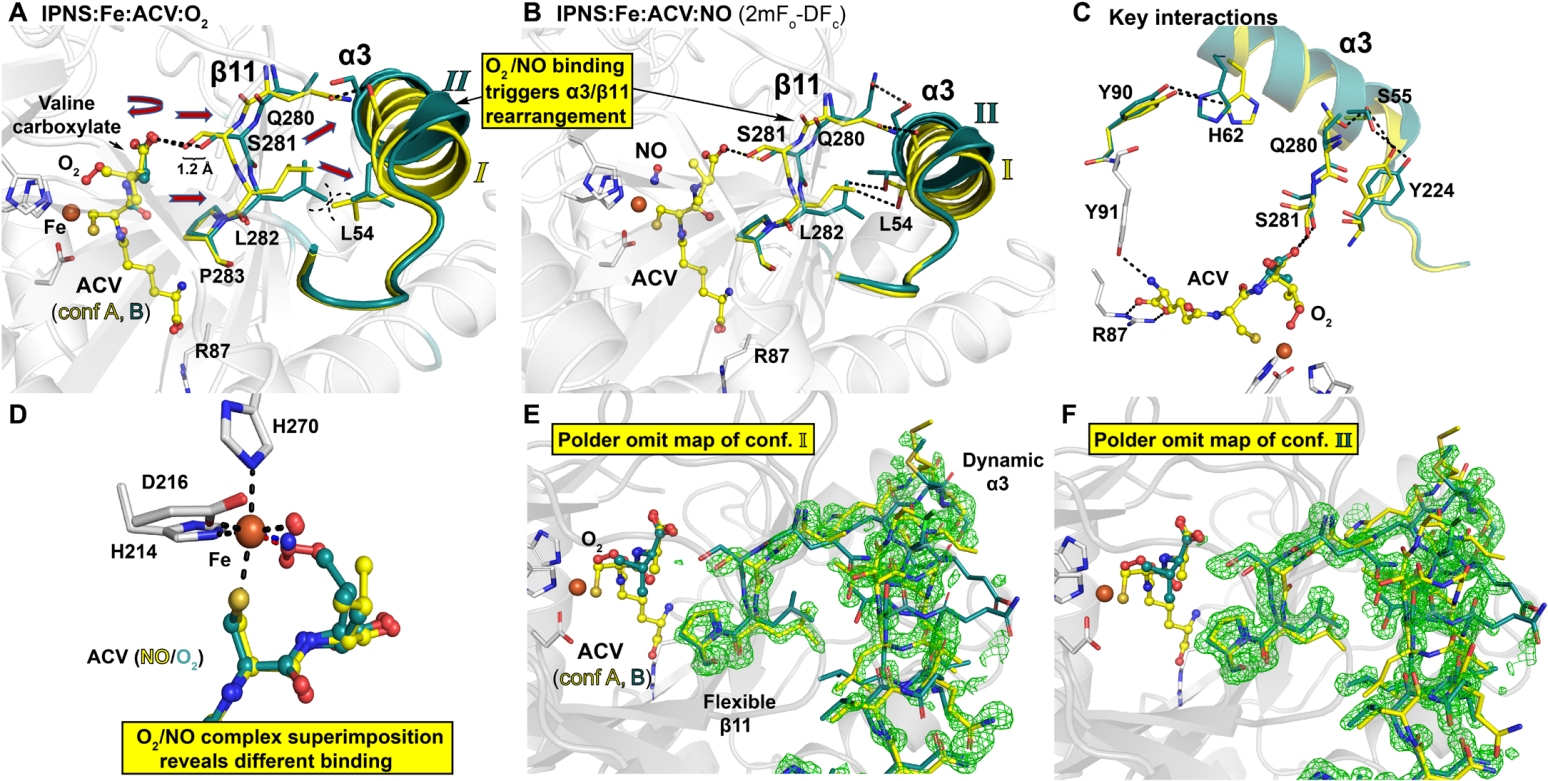
O_2_ binding to IPNS:Fe:ACV causes unexpected conformational changes. (**A**) Refined model for a cryo-cooled IPNS:Fe:ACV:O_2_ complex after O_2_ exposure (60 s) reveals Fe-O_2_ binding and induced rearrangements from confs. I to II of α3/β11 [PDB: 6ZAP, cryo MX, 1.36-Å resolution, yellow: conf. I (−O_2_); teal: conf. II (+O_2_)]. Ser^281^, which is involved in ACV valine carboxylate binding shifts by 1.2 Å. Movements are indicated by red arrows; hydrophobic interactions (L^282^_β11_/L^54^_α3_) by dashed lines (fig. S7). (**B**) The IPNS:Fe:ACV:NO (PDB: 6ZAN, 1.39-Å resolution) complex refines to two equal α3/β11 confs. I/II (fig. S7). (**C**) Hydrogen bond network involved in O_2_ binding induced α3/β11 rearrangements. (fig. S7). (**D**) Superimposition of both complexes reveals different NO and O_2_ binding modes [Fe-O_1_-O_2_ (135°) and Fe-N-O (130°); fig. S7]. (**E** and **F**) Polder omit maps (3.0 σ contour level) of residues involved in O_2_ binding–induced rearrangement carved around β11/α3, omitting (E) conf. I and (F) conf. II, showing confs. I and II of β11/α3, respectively.

B-factor analyses of the tr-SFX O_2_–exposed datasets reveals increased dynamics of α3; however, we were unable to confidently fit and refine discrete new conformations because the electron density maps indicated that these are present at low occupancy (10 to 15%). Therefore, we attempted to trap thermodynamically unstable conformations by exposing single IPNS:Fe:ACV crystals to O_2_ and then plunging them into liquid nitrogen for cryo-synchrotron analysis. Analysis of ~100 IPNS:Fe:ACV crystals exposed to O_2_ for times ranging from 30 to 600 s shows evidence for electron density corresponding to O_2_ binding trans to Asp^216^ [although photoelectric reduction likely occurs (*18*)] with a trend to disorder of the ACV valine, along with increased α10 and α3 B-factors. Notably, under the cryo-conditions, along with the starting conformations as observed previously (I), we unequivocally observed the formation of discrete new conformations for α3 and β11 (II) (Fig. 5 and fig. S7).

Similar changes in valine disorder, increased B-factors and the new α3/β11 conformations, were observed on formation of the IPNS:Fe:ACV:NO complex in single crystals (Fig. 5 and fig. S7). A structure of the IPNS:Fe:ACV:NO complex (PDB: 1BLZ) (*26*) has been reported; alternative confs. of α3 and β11 due to NO binding were not refined, possibly due to low occupancies of the flexible regions. As for the tr-SFX–observed O_2_ binding and as reported in a synchrotron derived structure (*26*), NO binds trans to Asp^216^, but with a slightly different orientation to O_2_ (Fig. 5D). These NO or O_2_-exposed structures were refined with both α3/β11 conformations (I and II)—the IPNS:Fe:ACV:NO complex and a representative IPNS:Fe:ACV:O_2_ complex with 1:1 occupancy of conformations I and II were selected for deposition. The difference between the cryo-condition work, where we observed the discrete new conformations for α3/β11, and the tr-SFX studies, where we did not accrue unequivocal evidence for them, likely in part reflects higher mobility under the latter room temperature conditions.

**Fig. 6:**
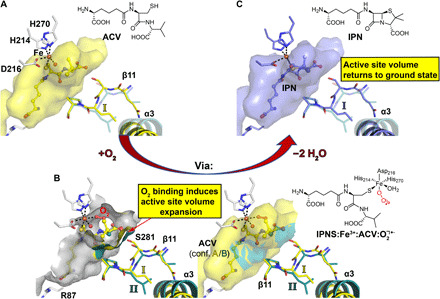
Comparison of active site volume before and after O_2_ binding–induced rearrangement of β11 and α3. Calculations of the active site volume were performed with PyVOL (*61*). (**A**) Active site volume presentation of the IPNS:Fe:ACV complex (yellow). The alternative conf. II obtained after O_2_ binding is in teal with high transparency. (**B**) Active site volume superimposition of the atomic model obtained from the single-crystal IPNS:Fe:ACV:O_2_ (PDB: 6ZAP) complex by cryo-cooled synchrotron data collection highlighting the two different conformations of β11 and α3 triggered by O_2_ binding. The two panels show the active site volume in an open (left) and closed (right) view. To perform active site volume calculations, either conf. A (ground state, yellow) or B (O_2_ bound, teal) was removed from the model. The active site volume after O_2_ binding (teal, 1374 Å^3^) appears to increase by 13 Å^3^ compared to conf. A, as obtained in the anaerobic ground state (yellow, 1361 Å^3^). Note that these changes appear solely in the region where the bond-breaking and bond-forming steps for penicillin formation are happening. (**C**) Active site volume presentation of the IPNS:Fe:IPN complex (blue). Since β11 in the IPNS:Fe:ACV and IPNS:Fe:IPN complexes superimpose identically (not shown), the active site volume returns back to its ground state after turnover. These observations suggest a controlled active site volume expansion during penicillin ring formation. The alternative conf. II obtained after O_2_ binding is shown in teal with high transparency.

The combined tr studies enable experimentally based proposals for the underlying interactions involved in dynamics during IPNS catalysis. Although motions other than those involving α3/β11, including the C-terminal region in substate binding and product release, are likely involved, our studies reveal a critical role for α3/β11 dynamics in β-lactam ring formation. O_2_ binding induces mobility of the ACV valine weakening its interaction with Ser^281^_β11_ causing β11 to move away from ACV (Fig. 5, fig. S7, and movie S1). This increases the active site volume by ~13 Å^3^, so enabling productive movement of the CV unit of ACV to initially form a thioaldehyde and then a monocyclic β-lactam (Fig. 6).

As a consequence of the movement of Ser^281^_β11_ and other β11 residues, conformational changes occur throughout α3 on the protein surface (amino acids 47 to 64, α3 51 to 64). The correlated motions involving α3/β11 are enabled by interactions between Gln^280^_β11_ and Ser^55^_α3_, Leu^282^_β11_ and Leu^54^_α3_, Tyr^224^_α6_ and Ser^55^_α3_ [Tyr^224^ is conserved and adjacent to Leu^223^, which is part of a hydrophobic pocket binding the ACV valine isopropyl (*26*, *33*)] and His^62^_α3_ and Tyr^90^_α3_ (Fig. 5C and fig. S7). Tyr^90^ is adjacent to Tyr^91^, which binds the ACV Aad amino group via a hydrogen bond (Fig. 5C). The Aad side chain, including its amino group, becomes disordered following O_2_ exposure but is ordered in the IPN product complex. α3 thus plays a central role in conformational changes involved in the conversion of ACV to IPN via dynamic interactions with both the ends of ACV/intermediates, i.e., with the Aad amino group and the Val carboxylate.

**Fig. 7:**
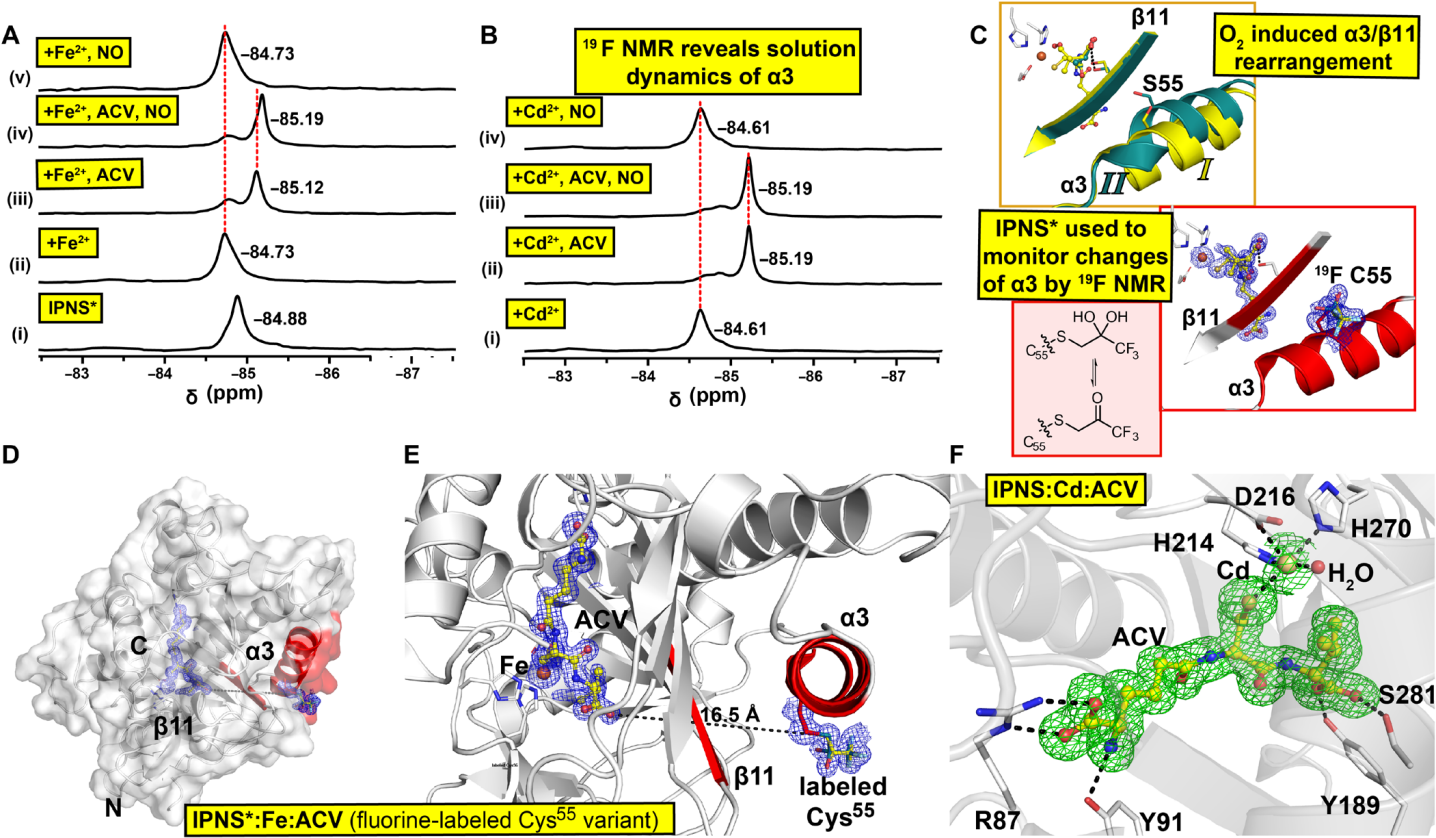
Spectroscopic analyses in solution support structural dynamics as a consequence of O_2_ binding. (**A**) ^19^F NMR spectra from anaerobic solutions of IPNS* ± Fe^2+^ ± ACV ± NO support α3 dynamics after NO binding. (i) IPNS* (^19^F-labeled IPNS); (ii) IPNS* + Fe^2+^; (iii) IPNS* + Fe^2+^+ACV; (iv) IPNS* + Fe^2+^ACV + NO; and (v) IPNS* + Fe^2+^+NO. (**B**) ^19^F NMR of IPNS* ± Cd^2+^ ± ACV ± NO. (i) IPNS* + Cd^2+^; (ii) IPNS* + Cd^2+^+ACV; (iii) IPNS* + Cd^2+^ACV + NO; and (iv) IPNS* + Cd^2+^+NO (fig. S8). (**C**) Comparison of the structural elements of α3/β11 in IPNS:Fe:ACV:O_2_ (PDB: 6ZAP) and IPNS*:Fe:ACV (cryo-cooled single-crystal analysis, PDB: 6ZAM, 1.55-Å resolution, used for ^19^F NMR, showing keto and gem diol forms of fluorine label). (**D**) Overview of the IPNS* complex with α3 and β11 in red and presence of the fluorine label on α3. (**E**) View of the active site and α3 of the IPNS*:Fe:ACV complex, showing the distance from the ACV valinyl carboxylate to the alkylated Cys^55^ (16.5 Å). Changes in the ACV valinyl carboxylate conformation as a function of O_2_ binding are proposed to induce structural rearrangement of α3/β11. Cys^55^ was alkylated with a CH_2_COCF_3_ group to study conformational changes in solution by ^19^F NMR. Tryptic digestion confirmed the presence of alkylated Cys^55^ (fig. S10). (**F**) Views of the active site of the aerobically grown IPNS:Cd:ACV complex (PDB: 6ZW8, 1.22-Å resolution) obtained by single-crystal cryogenic data collection. A Polder omit map of the active site of IPNS:Cd:ACV after omitting Cd and ACV (3.0 σ contour level, green, modeled occupancy of 90% for Cd). Unmodeled electron density was observed next to Trp^213^ on the protein surface. Note that exposure of IPNS:Cd:ACV crystals to NO reveals no NO binding to the metal and no evidence for α3/β11 rearrangements, even after multiple NO exposures with 10 crystals, consistent with the NMR analysis (B).

We then used ^19^F-NMR to investigate whether the conformational changes observed for α3 in crystals occur in solution, by monitoring changes of IPNS ^19^F-labeled (*34*) on α3 (IPNS*; Fig. 7 and fig. S8). The results reveal distinct signals for the apo, Fe(II), and Fe(II):ACV complexes. Addition of NO to IPNS*:Fe:ACV, but not IPNS*:Fe or IPNS*:Cd:ACV (Fig. 7 and fig. S8) complexes, resulted in movement of the IPNS* ^19^F signal (Fig. 7 and fig. S8), supporting the proposal that O_2_/NO binding induces α3 dynamics in presence of ACV, since NO should not bind to the Cd(II) complex.

## DISCUSSION

Our studies provide new insights how correlated motions induced by O_2_ binding enable IPNS to catalyze β-lactam formation, including by transiently altering the active site volume and arranging the substrate conformation in preparation for ring formation. Sequence comparisons reveal that α3/β11 and key residues involved in the associated correlated motions are conserved in IPNS ranging from prokaryotes to an animal (fig. S9A) (*35*). β11 (which extends one β sheet of the core DSBH fold) and α3 (one of the two conserved exterior N-terminal helices) are present in multiple 2OG oxygenases, ranging from those of cephalosporin biosynthesis which are closely related to IPNS to those involved in DNA/RNA repair, histone demethylation, lipid metabolism, and the human hypoxic response (fig. S9B) (*5*, *6*). Thus, although there are likely variations, and the extent of protein dynamics during catalysis is likely underestimated in crystallographic studies, the types of correlated motions observed by tr-SFX during IPNS catalysis are likely of widespread relevance. Detailed knowledge of how oxygenases work should enlighten research concerning their biological roles as sensors, on engineering them to alter the course of biosynthesis and on modulating their activity for therapeutic benefit.

The roles of dynamics in tuning catalysis of individual steps are of general interest in catalysis, particularly for enzymes that catalyze chemically challenging reactions involving substantial conformational changes (*36*). tr-SFX is a powerful method for uncovering these, including, as shown here, motions involved in steps after substrate binding and before product release. We hope increased knowledge of how IPNS works will promote the use of tr-SFX to study synthetically challenging reactions and inspire the discovery of new types of nonprotein catalysts making densely functionalized and conformationally strained ring systems. However, our results demonstrating that motions outside the active site are important in catalyzing conversion of a “simple” peptide to the penicillin nucleus suggest that such biomimetic catalysts may need to be macromolecular.

## MATERIALS AND METHODS

Chemicals for preparation of buffers and crystallization screens were from commercial suppliers and were used without further purification. ACV was synthesized by solid phase peptide synthesis and purified using a Shimadzu high-performance liquid chromatography (HPLC) system, equipped with a SunFire semiprep column (C18, 5 μm, 150-mm length, 10-mm diameter). The mass of ACV was confirmed by LC-MS; Agilent Technologies 1260 Infinity Series, equipped with a 6120 quadrupole mass spectrometer using a Merck Chromolith Performance C18 (100 mm by 4.2 mm) HPLC column. ACV purity was confirmed by NMR (Bruker AVIII HD 600 equipped with a BB-F/H N_2_ Prodigy CryoProbe). Recombinant wt-IPNS (pJB703) from *Aspergillus nidulans* in NM554 *Escherichia coli* was produced by the reported procedure (*37*).

### Cloning of pCOLD_IPNS

DNA (codon-optimized for expression in *E. coli*) of IPNS (GeneArt; Thermo Fisher Scientific, UK) was inserted into the pCOLD I vector (Addgene, UK) using Sal I and Not I restriction sites. This vector enabled production of IPNS with an *N*-terminal hexa-histidine tag (6xHis) with an *N*-terminal C human rhinovirus protease cleavage site as reported (*38*).

### Mutation of pCOLD_IPNS S55C

A Ser^55^ to Cys variant of codon-optimized IPNS produced with the pCOLD vector was obtained using the Q5 site–directed mutagenesis kit (New England Biolabs, USA) followed by DpnI digestion at 32°C for 5 hours. Primers used are as follows: IPNS_S55C_fwd: *cattaatgtgcagcgtctgtgccagaaaaccaaagaatt* and IPNS_S55C_rev: *aattctttggttttctggcacagacgctgcacattaatg*. The polymerase chain reaction product was transformed into XL10 Gold ultracompetent cells (Agilent Technologies, USA) by heat shock and grown overnight on 2YT agar plates containing ampicillin (50 μg ml^−1^). A single colony was picked and cultured in liquid 2YT media while shaking (150 rpm) overnight at 37°C. Plasmid DNA was isolated using the GeneJET Plasmid Miniprep kit (Thermo Fisher Scientific, USA). The presence of the desired mutation was confirmed by DNA sequencing (Eurofins, Germany).

### Expression and purification of IPNS_S55C and labeling of IPNS_S55C with BTFA (*34*, *39*)

The pCOLD_IPNS_S55C plasmid was transformed into *E. coli* BL21 cells and grown in 2YT agar plates containing ampicillin (50 μg ml^−1^). Single colonies were picked and cultured in 2YT media (100 ml), supplemented with ampicillin (50 μg ml^−1^), overnight with shaking (150 rpm, 37°C). A starter culture (6 ml; 1:100, v/v) was used to inoculate a large-scale growth in 2YT media (600 ml) supplemented with ampicillin (50 μg ml^−1^) with shaking (150 rpm, 37°C) until an appropriate OD_600_ (optical density at 600 nm) = 0.6 was reached. Expression was induced by β-d-1-thiogalactopyranoside (1 mM final concentration) addition; cultures were incubated overnight at 15°C. Cells were harvested by centrifugation (11,000*g*, 10 min, 4°C) and stored at −80°C.

A cell pellet (25 g) was resuspended (1:4 w:v) in buffer G [50 mM tris (pH 7.5), 200 mM NaCl, and 5 mM imidazole] supplemented with deoxyribonuclease I (10 μg ml^−1^), phenylmethylsulfonyl fluoride (10 μg ml^−1^), and lysozyme (0.2 mg ml^−1^) by stirring at 4°C and for 30 min. Cells were lysed by sonication (9 s on: 9 s off, 60% amplitude, 12-min total time, 4°C; Sonics Vibra-Cell), cell debris was removed by centrifugation (58,000g, 30 min, 4°C), and the supernatant was filtered (0.45 μm). The filtrate was loaded onto a nickel affinity column (5 ml HisTrap HP, GE Healthcare, USA), pre-equilibrated with buffer G (20 CV), and eluted with a gradient from buffer G to buffer H [50 mM tris (pH 7.5), 200 mM NaCl, 500 mM imidazole, and 20 CV]. Fractions containing IPNS were identified by SDS–polyacrylamide gel electrophoresis (PAGE) and concentrated by centrifugation [10,000 molecular weight cut-off (MWCO), 3000*g*, 4°C; Merck Millipore, USA], before purification by Superdex 75 (300 ml, GE Healthcare, USA) chromatography using a column pre-equilibrated with buffer I [25 mM tris (pH 7.5), 500 mM NaCl, and 1 CV]. Fractions containing protein were identified by ultraviolet analysis and analyzed by SDS-PAGE. Fractions containing purified IPNS_S55C were concentrated using a centrifugation tube (10,000 MWCO, 3000*g*, 4°C; Amicon Ultra). Removal of the *N*-terminal hexa-histidine affinity tag was achieved by incubation with 3C protease (1:100, w/w) overnight at 4°C. Successful cleavage was confirmed by protein MS (Quattro Premier XE, Waters, USA). Untagged IPNS_S55C was subsequently purified using a nickel affinity column (5 ml HisTrap HP, GE Healthcare, USA) using buffer G (5 CV). A solution of IPNS_S55C (350 μM) was incubated with tris(2-carboxyethyl)phosphine [350 μM, 1 equivalent (eq.)] in buffer J [25 mM tris (pH 8.5) and 100 mM NaCl] for 5 min at 4°C. 3-Bromo-1,1,1-trifluoro acetone (BTFA; 10.5 mM final concentration, 30 eq.) was added, and the solution was incubated (90 min) at 4°C. Alkylation of IPNS_S55C was confirmed by MS (Quattro Premier XE, Waters, USA); the observed mass for IPNS_S55C (37,768 Da) demonstrated an increase by 128 Da when alkylated to give IPNS* (37,896 Da, BTFA-labeled IPNS_S55C). Note that the predicted mass difference based on the calculated mass of CH_2_C(O)CF_3_ (110 Da) was higher by 18 Da than observed, suggesting that the ketone exists mainly in its hydrated form, as observed for a different protein (*34*, *40*, *41*), and consistent with our crystallization analyses (Fig. 7). The reaction was stopped by buffer exchange using PD-10 columns, pre-equilibrated with buffer J. The BTFA-labeled IPNS_S55C (IPNS*) variant was concentrated to a volume < 2 ml and further purified by size exclusion chromatography using a Superdex 75 column (300 ml, GE Healthcare, USA) pre-equilibrated with buffer J. Protein containing fractions were subsequently dialyzed as described above. The apo-IPNS* was concentrated to 50 mg ml^−1^, aliquoted, and stored at −80°C. Trypsin digestion was performed to confirm the labeling (fig. S10).

### Tryptic digestion

Tryptic digestion was performed according to a reported procedure (*42*). Labeled IPNS* (15 μl, 1 mg ml^−1^) was treated with dithiothreitol (DTT; 2 μl, 85 mM), in ammonium bicarbonate buffer (10 mM), for 40 min at 56°C. The protein was then alkylated with iodoacetamide (7 μl, 55 mM) for 30 min and then treated with DTT (3 μl, 85 mM) for 10 min at room temperature (rt) in the dark. Trypsin (3 μl; 1:20, w/w) was added and reacted for 16 hours at 37°C. The sample was then diluted with acetonitrile (120 μl; 1:100, v/v) and allowed to react for 3 hours at 37°C. Digestion was stopped by the addition of formic acid (7.5 μl), and the mixture was then vacuum centrifuged (Eppendorf, Germany) to dryness. The residue was redissolved in H_2_O (18 μl) supplemented with aqueous formic acid (0.1% v/v). After pre-equilibrating a ZipTip column (Merck Millipore, USA) with aqueous acetonitrile (98:2, 0.1% formic acid), the sample was aspirated into the ZipTip column. The fragments were eluted from the ZipTip column using aqueous acetonitrile (40:60, 0.1% aqueous formic acid), vacuum centrifuged to dryness and redissolved in aqueous acetonitrile (98:2, 0.1% formic acid), and analyzed by nanoLC (Thermo Elite, Thermo Fisher Scientific, USA), and data were analyzed using BSI PEAKS studio 8.5. The results from the trypsin digestion are shown in fig. S10.

### Nitric oxide exposure of IPNS crystals and solutions

Experiments involving nitric oxide (NO) were carried out by two researchers using a fume hood. A two-chambered, four-valve glass apparatus (fig. S11) was used for controlled NO exposure, minimizing O_2_ and user exposure. Anaerobic protein/crystal samples, sealed in J Young valve NMR tubes (5 mm, Norell, USA), were attached to the NMR valve without opening the NMR tube (fig. S11). The system was first purged with argon (10 min) with the gas outlet and separating valves open. The gas outlet valves were closed, under a slight overpressure of argon. Chamber 2 was sealed by closing the separating valve, and vacuum was applied by opening the chamber 2 outlet valve. To create a mild vacuum in the NMR tube, the outlet valve was closed before opening the NMR valve. After equilibration (~2 min), the NMR valve was closed and the chamber 2 outlet valve was reopened. This process was repeated twice. Chamber 1 was purged with NO [1000 parts per million (ppm) in nitrogen, (0.1% NO and 99.9% N_2_), 10 min; BOC, UK]. After purging chamber 1, the outlet valve was closed while the system was under a positive pressure, and the balloon was partially filled with NO. To expose the sample to NO, the separating and NMR valves were opened. Samples were incubated with NO for 10 to 30 min to yield a pale pink solution (*12*) in the NMR tube. The NMR valve was closed and samples was removed for analysis. To remove NO from the system, all valves were opened and flushed with argon for several minutes. Note that, for safety reasons, no vacuum was applied once the system was filled with NO.

### NMR studies

NMR experiments on IPNS* [the ^19^F-BTFA–labeled IPNS_S55C mutant isoform (trifluoroacetonyl labeled); Fig. 7 and fig. S8] were recorded using a Bruker AVIII HD 600 equipped with a BB-F/H ProdigyN_2_ CryoProbe, in 5-mm regular or J Young valve NMR tube (5 mm; Norell, USA), at 298 K, unless stated otherwise. ^19^F NMR spectra were referenced to CF_3_CO_2_H (100 μM, at δ_F_ = −76.55 ppm) and processed with 3- or 30-Hz Lorentzian line broadening using MestReNova 14.1 (MestReLabs, Spain; www.mestrelab.com) and TopSpin 3.6.1 (Bruker, Germany; www.bruker.com). Sample preparation was performed under anaerobic conditions (<2-ppm O_2_) in an anaerobic chamber (Belle Technology, UK). Solutions [in 25 mM tris-d_11_ buffer (pH 7.5)] were deoxygenated by argon purging (30 min) before placing in an anaerobic chamber. Solids (FeSO_4_ and ACV) and NMR tubes were transferred into the glovebox and left to equilibrate (16 hours). Solutions of apo-IPNS* (50 mg ml^−1^, 1.35 mM, and 50 μl) were transferred to the anaerobic chamber immediately before use. Appropriate stock solutions of ACV (10 mM), FeSO_4_ (100 mM), and CF_3_CO_2_H (10 mM) were prepared in a glovebox. The total volume of a typical sample was 450 μl and contained 10% (v/v) D_2_O. After equilibration, the sample was transferred from an Eppendorf tube (1.5 ml) into a 5-mm J Young valve NMR tube (Norell, USA) for analysis.

For the addition of subsequent solutions to the sample, a J Young valve NMR tube was transferred into the glovebox and equilibrated for 5 to 10 min before opening. The appropriate solution was added, and the tube was closed and inverted to equilibrate. For exposing a sample inside a J Young valve NMR tube to nitric oxide (NO), the tube was connected to an appropriate glass apparatus (fig. S11), and the sample was treated as described above. The J Young valve was closed before disconnecting the tube and inverted carefully several times to allow mixing of the gas in the headspace with the solution.

### Preparation of salt free IPNS and nondenaturing MS

Purified IPNS was used for nondenaturing electrospray ionization MS after buffer exchange into ammonium acetate (1 M). Buffer exchange was performed using Zeba Micro Spin desalting columns (Thermo Fisher Scientific) followed by 3-hour dialysis with ammonium acetate (1 M). Samples were mixed with the different (co-)substrates dissolved in Milli-Q after different incubation times. Nondenaturing MS data were acquired using an Orbitrap extended mass range prototype (*43*) machine. Data were recorded in positive ion mode, from a static nanospray source, using a gold-plated capillary prepared in house. Nitrogen was used as a collision gas [the pressure of around 1 × 10^−9^ mbar recorded in the Orbitrap, no HCD voltage (0 V) applied]. The capillary temperature was set to 30°C, and a spray voltage of 1.4 kV was applied. Acquired data were processed and analyzed using Thermo Xcalibur 4.13 (Thermo Fisher Scientific, UK) (*43*).

### Optimization of microcrystals

Crystallization was conducted within an anaerobic chamber maintained at 2 ppm or less O_2_ (Belle Technologies, UK) with plates, solutions, and other equipment used for crystallization deoxygenated within the chamber for at least 24 hours. The IPNS solution that was used to grow crystals was deoxygenated in the anaerobic chamber for 1 to 2 hours before use.

IPNS microcrystals were prepared as reported (*27*). For setting up a batch plate of microcrystals, IPNS (200 μl, *c* = 52 mg ml^−1^), ACV (2.2 mg), FeSO_4_·7H_2_O (1 eq., 100 mM stock in H_2_O, 2.78 μl) and seed stock solution (12 μl) were transferred into an anaerobic chamber (Belle Technologies, UK). Using a 96-well plate (Corning, USA), solutions for the batch setup were prepared as follows. For each well, Li_2_SO_4_ (66.5 μl of 2.7 M in H_2_O), H_2_O (13.5 μl), tris buffer (10 μl of 1.0 M in H_2_O, pH 8.5), and IPNS mixture (6.5 μl) were required. For one aliquot of IPNS solution (200 μl, 50 mg ml^−1^, 1.4 mM), a solution for 30 wells comprising Li_2_SO_4_ (1.995 ml, 2.7 M in Milli-Q), Milli-Q (405 μl), and tris [300 μl, 1.0 M in Milli-Q (pH 8.5)] was prepared. Before addition of IPNS to the crystallization solution, a freshly prepared FeSO_4_ solution (2.8 μl, 1.0 equivalent, 100 mM in Milli-Q water) was added to the IPNS solution; the mixture was equilibrated for 10 min. ACV (2.2 mg) was dissolved in tris buffer [40 μl, 25 mM in Milli-Q (pH 8.5)] and added in 4 × 10 μl portions carefully to the protein solution; precipitation was removed by centrifugation. The clear protein solution (containing ACV and FeSO_4_, ~220 μl) was added to the crystallization solution. The seed stock (12 μl) was then added and the mixture was pipetted into the wells (100 μl). The plate was carefully sealed (Polyolefin StarSeal, Starlab, UK) and shaken (700 rpm) using a microplate shaker (SciQuip, UK) for 16 to 30 hours depending on the temperature and the relative humidity in the anaerobic chamber. The appropriate shaking speed enabled production of needle morphology microcrystals (1 ml, *c* = 2 × 10^7^ microcrystals/ml) of homogeneous size typically with 3 μm × 3 μm × (40 to 60) μm size range (fig. S12).

### Single-crystal preparation methods for MX: IPNS*:Fe:ACV and IPNS:Cd:ACV

Purified recombinant *apo*-IPNS* or *apo*-IPNS was used for crystallization. IPNS crystals were grown in 24-well hanging drop VDX plates (Hampton Research, USA), anaerobically for the IPNS*:Fe:ACV and aerobically for the IPNS:Cd(II):ACV complex. The IPNS crystallization solution was prepared by mixing freshly prepared FeSO_4_ or CdCl_2_ (4 μl, 100 mM) with IPNS [in 25 mM tris (pH 8.0), 80 μl of 50 to 52 mg ml^−1^], followed by a subsequent addition of ACV [4 × 5 μl, 2.1 mg in 20 μl of 25 mM tris (pH 8.5)]. A screen, varying the pH [0.1 M tris (pH 8.1 to 8.7 in steps of 0.2), vertical axis] and the salt concentration (Li_2_SO_4_ 1.5 to 2.0 M in steps of 0.1, horizontal axis), was carried out (*28*). Crystals were prepared using the hanging drop method by combining the reservoir solutions (3 μl) and protein solutions (3 μl). Crystals (60 to 200 μm) formed after 24 to 36 hours were harvested and cryo-cooled by rapid plunging into liquid N_2_ before data collection. Single IPNS crystals were cryoprotected by transferring to a solution of mother liquor [1.7 M Li_2_SO_4_ and 0.1 M tris (pH 8.5)] supplemented with 20% (v/v) glycerol before being cryo-cooled in liquid N_2_. Data for the single crystals were collected at 100 K using synchrotron radiation at the Diamond Light Source beamlines I03, I04, I04-1, and I24 and processed using the Xia2 pipelines (table S1) (*44*).

Note that formation of IPNS:Cd:ACV crystals under aerobic conditions was only possible when the starting enzyme was completely metal ion free, possibly due to ACV turnover by a small amount of Fe(II)-bound enzyme in solution under aerobic conditions. Needle-shaped crystals were used to prepare seeds using the PTFE Seed Bead Kit as described by the manufacturer (Hampton Research, USA). These crystals were used in batch methods (as described above) to obtain more IPNS:Cd:ACV crystals for NO exposure experiments. Note that while all complexes crystallized with Fe(II) show clear turnover in crystallo and in solution (followed by NMR and mass spectroscopy) (*33*, *45*) after removal from the anaerobic chamber and exposure to O_2_ from air, the Cd(II) complex crystallized under aerobic conditions and showed no turnover of ACV to IPN.

### Setup and sample injection for crystallographic data collection

Room temperature diffraction data for microcrystal slurries were collected at the MFX (Macromolecular Femtosecond Crystallography) instrument of Linac Coherent Light Source (LCLS) (*46*, *47*) (proposals LU50/P143 and LS34/P110) and at the BL2 beamline at SACLA (proposal 2017B8085) (table S1). The drop-on-tape (DOT) sample delivery method (*23*) was used combined with ADE at LCLS to obtain anaerobic IPNS:Fe(II):ACV complex and tr O_2_–exposed structures (*23*). The ejected droplets (~3.5 to 4 nl; flow rate, 7 ml min^−1^) are deposited onto the conveyor belt at room temperature in a helium atmosphere. To trigger the reaction in crystallo, our method exposes the microdroplets of the anaerobic crystal slurry to a 100% O_2_ atmosphere as they pass through a 60-mm-long reaction chamber for a varied time regulated by the Kapton belt speed (table S1) (*23*). We collected tr-SFX datasets after exposing droplets to O_2_ and then applying additional reaction times of 400, 500, 800, 1600, and 3000 ms. Shorter reaction times derive from faster belt speeds, which reduces the overall time for O_2_ diffusion into the microdroplets and hence reaction within the microcrystals. Longer reaction times derive from a slower belt speed and commensurately longer O_2_ droplet equilibration and reaction times. Detailed information about the Kapton tape speed (millimeter per second) and the O_2_ incubation times (millisecond, including incubation time in the reaction chamber and additional travel time of microdroplets from the reaction chamber until they arrive in the x-ray interaction zone) are shown in table S1. The x-ray wavelength for experiments obtained under proposals LU50/P143 was kept at 1.30 Å (9.537 keV) and LS34/P110 at 1.31 Å (9.464 keV) with a data collection rate of 30 Hz, 4 mJ/pulse, a pulse duration of ~35 fs and an x-ray beam size at the sample of ~3 μm in diameter. XRD data were collected using a Rayonix MX340-HS detector for the LU50/P143 beam time and on a Rayonix MX170-HS detector for LS34/P110 with 4 × 4 binning.

The IPNS:Fe:IPN product complex SFX data were collected using the viscous extruder and a grease-matrix carrier by first exposing anaerobic slurries of the IPNS:Fe(II):ACV complex (crystal density > 5 × 10^7^ ml^−1^) to atmospheric O_2_ for 30 min and then mixing the slurry with grease in the ratio 10 μl slurry:90 μl grease at SACLA (*48*). Mixing of grease with slurry was performed by using two 100-μl Hamilton syringes connected over an extruder-based connector. The obtained grease matrix containing randomly oriented protein microcrystals was applied to the sample reservoir (60 μl of reservoir and 2 mm in diameter) of the sample delivery system and mounted in the setup for data collection. A flow rate of 1 to 1.5 μl min^−1^ and a nozzle dimension of 100 μm were used to obtain a stable flow for the protein grease matrix. Data were collected at room temperature with a collection rate of 30 Hz and an x-ray beam size at the sample of ~1.7 μm in diameter. The x-ray wavelength for the experiment was kept at 1.14 Å (11 keV) with 0.34 mJ/pulse and 10-fs pulse length. XRD data were collected on a MPCCD (MultiPort Charge-Coupled Device) octal detector.

### Data processing, model building, and refinement of SFX datasets

During the SFX experiments, data acquisition was tracked with the cctbx.xfel graphical user interface (*49*), which monitors for new data and submits processing jobs to the computing cluster. The jobs run the core program dials.stills_process to index and integrate the images while providing real-time feedback, as part of the larger cctbx.xfel and DIALS processing suite (*50*–*55*). A first estimate of the detector position (distance and beam center) was obtained from a powder diffraction pattern of silver(I) behenate (Alfa Aesar). After initial spot finding of strong reflections, followed by indexing of strong reflections and integration using dials.stills_process, a round of metrology refinement was done (*56*). A second round of indexing and integration was performed with the refined detector position. Initial merging was performed using PRIME (*57*) followed by molecular replacement with PHASER (*58*). This provided a reference model using a target unit cell of *a* = 41.9 Å, *b* = 75.7 Å, *c* = 102 Å, α = β = γ = 90° (space group *P* 2_1_2_1_2_1_). After correction of the integrated intensities for absorption by the Kapton conveyor belt, final data integration and merging were performed using cxi.merge (*21*). Justification of the resolution cutoff for the merged data was determined on the basis of multiplicity in the highest-resolution shell (>10-fold) and on CC_1/2_ (monotonic decrease) (see tables at the end of the Supplementary Materials with the merging statistics justifying the resolution cutoff) (*20*, *23*).

Structures were solved by isomorphous molecular replacement using the reported structural data file of IPNS [PDB: 1BLZ (*26*)] as a search model. All structures were iteratively fitted and refined using PHENIX (*59*) and Coot (*60*). Processing and refinement statistics for all anaerobically and O_2_-exposed IPNS structures are given in table S2.

### XES analysis

The on-demand ADE/tape drive sample delivery setup enables the simultaneous collection of tr-XES and tr-SFX (*23*) from the same sample and x-ray pulse to study the Fe oxidation state. We used a wavelength-dispersive von Hamos spectrometer with four cylindrically bent (*R* = 250 mm) germanium (440) crystals orthogonal to the sample with the center of the crystals located at 75.41° with respect to the interaction point and each focusing the emitted Fe Kα_1,2_ x-ray photons onto an ePix100 detector located below the sample x-ray interaction region. Calibration was performed using aqueous solutions of 10 mM Fe(III)(NO_3_)_3_ and Fe(II)Cl_2_ (anaerobically prepared and measured) as references. Detector images were sorted by the sample hit rate as described (*25*). Briefly, for anaerobic, 400-, 500-, 800-, and 1600-ms datasets, two threshold values were set as 3 and 2 for thresholds I and II, respectively. For the 3000-ms dataset, the value for threshold II, which compares the number of photons per unit area inside and outside the region of interest, was reduced to 1. A linear function interpolated across two points outside the region of interest was subtracted from the images for background correction. For spectral analysis, the spectra were area-normalized in the 6380- to 6415-eV range. In this range, the recorded energy was initially spread out by 0.146- to 0.159-eV intervals. To create equal intervals and apply smoothing, the spectra were first linearly splined to a 0.001-eV interval and then processed by a second-order Savitzky-Golay filter with a 3003-point window size. This window size covers approximately the same range of points as a 19-point window applied to the original intervals. Difference spectra were calculated after smoothing. For calculation of FWHM, the spectral range for Kα_1_ was selected as 6396 to 6407 eV. Error bars for the calculated FWHM were obtained by a bootstrapping procedure as described (*25*).

### Exposure of anaerobically obtained single IPNS:Fe:ACV crystals to O_2_

IPNS:Fe:ACV crystals (needle morphology) were grown anaerobically to the appropriate size [~5 × 5 × (100 to 150) μm^3^] (*27*); the anaerobic microcrystal slurry (10 μl) was transferred into a 500-μl tube. A second 500-μl tube was filled with the IPNS crystallization buffer [100 μl, 1.7 M Li_2_SO_4_, and 0.1 M tris (pH 8.5) supplemented with 20% glycerol (v/v)], and the solution was saturated with O_2_. The anaerobic crystal slurry (2 μl) was removed from the glovebox and immediately mixed with the O_2_-saturated crystallization solution (8 μl). The samples were incubated at room temperature and at varying timepoints (30 s to 10 min). Single crystals were mounted on nylon loops and then cryo-cooled by rapid plunging into liquid N_2_. Data for a total of ~100 cryo-cooled single crystals of IPNS:Fe:ACV exposed to O_2_ were collected at the MX beamlines of the Diamond Light Source, UK. From the IPNS:Fe:ACV:O_2_ datasets, one representative structure was selected for deposition (PDB: 6ZAP), which was refined to 1.36-Å resolution. In this complex, both confs. A and B of α3 are observed and were refined in 50:50 occupancy.

## SUPPLEMENTARY MATERIALS

Supplementary material for this article is available at http://advances.sciencemag.org/cgi/content/full/7/34/eabh0250/DC1

## References

[1] H. R. Arnstein, D. Morris, The structure of a peptide, containing α-aminoadipic acid, cystine and valine, present in the mycelium of *Penicillium chrysogenum*. Biochem. J. 76, 357–361 (1960). 13794420 10.1042/bj0760357 PMC1204717

[2] N. I. Burzlaff, P. J. Rutledge, I. J. Clifton, C. M. H. Hensgens, M. Pickford, R. M. Adlington, P. L. Roach, J. E. Baldwin, The reaction cycle of isopenicillin N synthase observed by X-ray diffraction. Nature 401, 721–724 (1999). 10537113 10.1038/44400

[3] P. L. Roach, I. J. Clifton, V. Fülöp, K. Harlos, G. J. Barton, J. Hajdu, I. Andersson, C. J. Schofield, J. E. Baldwin, Crystal structure of isopenicillin N synthase is the first from a new structural family of enzymes. Nature 375, 700–704 (1995). 7791906 10.1038/375700a0

[4] P. Rabe, J. Kamps, C. J. Schofield, C. T. Lohans, Roles of 2-oxoglutarate oxygenases and isopenicillin N synthase in β-lactam biosynthesis. Nat. Prod. Rep. 35, 735–756 (2018). 29808887 10.1039/c8np00002f PMC6097109

[5] C. Q. Herr, R. P. Hausinger, Amazing diversity in biochemical roles of Fe(II)/2-oxoglutarate oxygenases. Trends Biochem. Sci. 43, 517–532 (2018). 29709390 10.1016/j.tibs.2018.04.002 PMC6014900

[6] C. J. Schofield, R. P. Hausinger, *2-Oxoglutarate-Dependent Oxygenases*, C. J. Schofield, R. P. Hausinger, Eds. (The Royal Society of Chemistry, 2015), pp. 1–487.

[7] R. Chowdhury, I. K. H. Leung, Y. M. Tian, M. I. Abboud, W. Ge, C. Domene, F. X. Cantrelle, I. Landrieu, A. P. Hardy, C. W. Pugh, P. J. Ratcliffe, T. D. W. Claridge, C. J. Schofield, Structural basis for oxygen degradation domain selectivity of the HIF prolyl hydroxylases. Nat. Commun. 7, 12673 (2016). 27561929 10.1038/ncomms12673 PMC5007464

[8] C. J. Schofield, P. J. Ratcliffe, Oxygen sensing by HIF hydroxylases. Nat. Rev. Mol. Cell Biol. 5, 343–354 (2004). 15122348 10.1038/nrm1366

[9] J. E. Baldwin, M. Bradley, Isopenicillin N synthase: Mechanistic studies. Chem. Rev. 90, 1079–1088 (1990).

[10] E. Tamanaha, B. Zhang, Y. Guo, W. C. Chang, E. W. Barr, G. Xing, J. St. Clair, S. Ye, F. Neese, J. M. Bollinger Jr., C. Krebs, Spectroscopic evidence for the two C-H-Cleaving Intermediates of *Aspergillus nidulans* Isopenicillin N Synthase. J. Am. Chem. Soc. 138, 8862–8874 (2016). 27193226 10.1021/jacs.6b04065 PMC4956533

[11] C. D. Brown, M. L. Neidig, M. B. Neibergall, J. D. Lipscomb, E. I. Solomon, VTVH-MCD and DFT studies of thiolate bonding to [FeNO]_7_/[FeO_2_]_8_ complexes of isopenicillin N synthase: Substrate determination of oxidase versus oxygenase activity in nonheme Fe enzymes. J. Am. Chem. Soc. 129, 7427–7438 (2007). 17506560 10.1021/ja071364v PMC2536647

[12] V. J. Chen, A. M. Orville, M. R. Harpel, C. A. Frolik, K. K. Surerus, E. Münck, J. D. Lipscomb, Spectroscopic studies of isopenicillin N synthase. A mononuclear nonheme Fe^2+^ oxidase with metal coordination sites for small molecules and substrate. J. Biol. Chem. 264, 21677–21681 (1989). 2557336

[13] M. Lundberg, P. E. Siegbahn, K. Morokuma, The mechanism for isopenicillin N synthase from density-functional modeling highlights the similarities with other enzymes in the 2-His-1-carboxylate family. Biochemistry 47, 1031–1042 (2008). 18163649 10.1021/bi701577q

[14] M. N. Blakely, M. A. Dedushko, P. Chaau Yan Poon, G. Villar-Acevedo, J. Kovacs, Formation of a reactive, alklyl thiolate-ligated FeIII-superoxo intermediate derived from dioxygen. J. Am. Chem. Soc. 141, 1867–1870 (2019). 30661357 10.1021/jacs.8b12670 PMC6942688

[15] M. Lundberg, K. Morokuma, Protein environment facilitates O_2_ binding in non-heme iron enzyme. An insight from ONIOM calculations on isopenicillin N synthase (IPNS). J. Phys. Chem. B 111, 9380–9389 (2007). 17637052 10.1021/jp071878g

[16] M. Lundberg, T. Kawatsu, T. Vreven, M. J. Frisch, K. Morokuma, Transition states in a protein environment—ONIOM QM:MM Modeling of Isopenicillin N Synthesis. J. Chem. Theory Comput. 5, 222–234 (2009). 26609836 10.1021/ct800457g

[17] C. D. Brown-Marshall, A. R. Diebold, E. I. Solomon, Reaction coordinate of isopenicillin N synthase: Oxidase versus oxygenase activity. Biochemistry 49, 1176–1182 (2010). 20078029 10.1021/bi901772w PMC2838496

[18] M. Unno, H. Chen, S. Kusama, S. Shaik, M. Ikeda-Saito, Structural characterization of the fleeting ferric peroxo species in myoglobin: Experiment and theory. J. Am. Chem. Soc. 129, 13394–13395 (2007). 17929929 10.1021/ja076108x

[19] K. Nass, A. Gorel, M. M. Abdullah, A. V. Martin, M. Kloos, A. Marinelli, A. Aquila, T. R. M. Barends, F. J. Decker, R. Bruce Doak, L. Foucar, E. Hartmann, M. Hilpert, M. S. Hunter, Z. Jurek, J. E. Koglin, A. Kozlov, A. A. Lutman, G. N. Kovacs, C. M. Roome, R. L. Shoeman, R. Santra, H. M. Quiney, B. Ziaja, S. Boutet, I. Schlichting, Structural dynamics in proteins induced by and probed with X-ray free-electron laser pulses. Nat. Commun. 11, 1814 (2020). 32286284 10.1038/s41467-020-15610-4 PMC7156470

[20] J. Kern, R. Chatterjee, I. D. Young, F. D. Fuller, L. Lassalle, M. Ibrahim, S. Gul, T. Fransson, A. S. Brewster, R. Alonso-Mori, R. Hussein, M. Zhang, L. Douthit, C. de Lichtenberg, M. H. Cheah, D. Shevela, J. Wersig, I. Seuffert, D. Sokaras, E. Pastor, C. Weninger, T. Kroll, R. G. Sierra, P. Aller, A. Butryn, A. M. Orville, M. Liang, A. Batyuk, J. E. Koglin, S. Carbajo, S. Boutet, N. W. Moriarty, J. M. Holton, H. Dobbek, P. D. Adams, U. Bergmann, N. K. Sauter, A. Zouni, J. Messinger, J. Yano, V. K. Yachandra, Structures of the intermediates of Kok’s photosynthetic water oxidation clock. Nature 563, 421–425 (2018). 30405241 10.1038/s41586-018-0681-2 PMC6485242

[21] I. D. Young, M. Ibrahim, R. Chatterjee, S. Gul, F. D. Fuller, S. Koroidov, A. S. Brewster, R. Tran, R. Alonso-Mori, T. Kroll, T. Michels-Clark, H. Laksmono, R. G. Sierra, C. A. Stan, R. Hussein, M. Zhang, L. Douthit, M. Kubin, C. de Lichtenberg, L. Vo Pham, H. Nilsson, M. H. Cheah, D. Shevela, C. Saracini, M. A. Bean, I. Seuffert, D. Sokaras, T. C. Weng, E. Pastor, C. Weninger, T. Fransson, L. Lassalle, P. Bräuer, P. Aller, P. T. Docker, B. Andi, A. M. Orville, J. M. Glownia, S. Nelson, M. Sikorski, D. Zhu, M. S. Hunter, T. J. Lane, A. Aquila, J. E. Koglin, J. Robinson, M. Liang, S. Boutet, A. Y. Lyubimov, M. Uervirojnangkoorn, N. W. Moriarty, D. Liebschner, P. V. Afonine, D. G. Waterman, G. Evans, P. Wernet, H. Dobbek, W. I. Weis, A. T. Brunger, P. H. Zwart, P. D. Adams, A. Zouni, J. Messinger, U. Bergmann, N. K. Sauter, J. Kern, V. K. Yachandra, J. Yano, Structure of photosystem II and substrate binding at room temperature. Nature 540, 453–457 (2016). 27871088 10.1038/nature20161 PMC5201176

[22] C. Gisriel, J. Coe, R. Letrun, O. M. Yefanov, C. Luna-Chavez, N. E. Stander, S. Lisova, V. Mariani, M. Kuhn, S. Aplin, T. D. Grant, K. Dörner, T. Sato, A. Echelmeier, J. Cruz Villarreal, M. S. Hunter, M. O. Wiedorn, J. Knoska, V. Mazalova, S. Roy-Chowdhury, J. H. Yang, A. Jones, R. Bean, J. Bielecki, Y. Kim, G. Mills, B. Weinhausen, J. D. Meza, N. al-Qudami, S. Bajt, G. Brehm, S. Botha, D. Boukhelef, S. Brockhauser, B. D. Bruce, M. A. Coleman, C. Danilevski, E. Discianno, Z. Dobson, H. Fangohr, J. M. Martin-Garcia, Y. Gevorkov, S. Hauf, A. Hosseinizadeh, F. Januschek, G. K. Ketawala, C. Kupitz, L. Maia, M. Manetti, M. Messerschmidt, T. Michelat, J. Mondal, A. Ourmazd, G. Previtali, I. Sarrou, S. Schön, P. Schwander, M. L. Shelby, A. Silenzi, J. Sztuk-Dambietz, J. Szuba, M. Turcato, T. A. White, K. Wrona, C. Xu, M. H. Abdellatif, J. D. Zook, J. C. H. Spence, H. N. Chapman, A. Barty, R. A. Kirian, M. Frank, A. Ros, M. Schmidt, R. Fromme, A. P. Mancuso, P. Fromme, N. A. Zatsepin, Membrane protein megahertz crystallography at the European XFEL. Nat. Commun. 10, 5021 (2019). 31685819 10.1038/s41467-019-12955-3 PMC6828683

[23] F. D. Fuller, S. Gul, R. Chatterjee, E. S. Burgie, I. D. Young, H. Lebrette, V. Srinivas, A. S. Brewster, T. Michels-Clark, J. A. Clinger, B. Andi, M. Ibrahim, E. Pastor, C. de Lichtenberg, R. Hussein, C. J. Pollock, M. Zhang, C. A. Stan, T. Kroll, T. Fransson, C. Weninger, M. Kubin, P. Aller, L. Lassalle, P. Bräuer, M. D. Miller, M. Amin, S. Koroidov, C. G. Roessler, M. Allaire, R. G. Sierra, P. T. Docker, J. M. Glownia, S. Nelson, J. E. Koglin, D. Zhu, M. Chollet, S. Song, H. Lemke, M. Liang, D. Sokaras, R. Alonso-Mori, A. Zouni, J. Messinger, U. Bergmann, A. K. Boal, J. M. Bollinger Jr., C. Krebs, M. Högbom, G. N. Phillips Jr., R. D. Vierstra, N. K. Sauter, A. M. Orville, J. Kern, V. K. Yachandra, J. Yano, Drop-on-demand sample delivery for studying biocatalysts in action at X-ray free-electron lasers. Nat. Methods 14, 443–449 (2017). 28250468 10.1038/nmeth.4195 PMC5376230

[24] J. Kern, R. Alonso-Mori, R. Tran, J. Hattne, R. J. Gildea, N. Echols, C. Glockner, J. Hellmich, H. Laksmono, R. G. Sierra, B. Lassalle-Kaiser, S. Koroidov, A. Lampe, G. Han, S. Gul, D. DiFiore, D. Milathianaki, A. R. Fry, A. Miahnahri, D. W. Schafer, M. Messerschmidt, M. M. Seibert, J. E. Koglin, D. Sokaras, T. C. Weng, J. Sellberg, M. J. Latimer, R. W. Grosse-Kunstleve, P. H. Zwart, W. E. White, P. Glatzel, P. D. Adams, M. J. Bogan, G. J. Williams, S. Boutet, J. Messinger, A. Zouni, N. K. Sauter, V. K. Yachandra, U. Bergmann, J. Yano, Simultaneous femtosecond X-ray spectroscopy and diffraction of photosystem II at room temperature. Science 340, 491–495 (2013). 23413188 10.1126/science.1234273 PMC3732582

[25] T. Fransson, R. Chatterjee, F. D. Fuller, S. Gul, C. Weninger, D. Sokaras, T. Kroll, R. Alonso-Mori, U. Bergmann, J. Kern, V. K. Yachandra, J. Yano, X-ray emission spectroscopy as an in situ diagnostic tool for X-ray crystallography of metalloproteins using an X-ray free-electron laser. Biochemistry 57, 4629–4637 (2018). 29906115 10.1021/acs.biochem.8b00325 PMC6081253

[26] P. L. Roach, I. J. Clifton, C. M. H. Hensgens, N. Shibata, C. J. Schofield, J. Hajdu, J. E. Baldwin, Structure of isopenicillin N synthase complexed with substrate and the mechanism of penicillin formation. Nature 387, 827–830 (1997). 9194566 10.1038/42990

[27] P. Rabe, J. H. Beale, A. Butryn, P. Aller, A. Dirr, P. A. Lang, D. N. Axford, S. B. Carr, T. M. Leissing, M. A. McDonough, B. Davy, A. Ebrahim, J. Orlans, S. L. S. Storm, A. M. Orville, C. J. Schofield, R. L. Owen, Anaerobic fixed-target serial crystallography. IUCrJ 7, 901–912 (2020). 10.1107/S2052252520010374 PMC7467159 32939282

[28] P. L. Roach, I. J. Clifton, C. M. Hensgens, N. Shibata, A. J. Long, R. W. Strange, S. S. Hasnain, C. J. Schofield, J. E. Baldwin, J. Hajdu, Anaerobic crystallisation of an isopenicillin N synthase∙Fe(II)∙substrate complex demonstrated by X-ray studies. Eur. J. Biochem. 242, 736–740 (1996). 9022704 10.1111/j.1432-1033.1996.0736r.x

[29] H. Nogami, Y. Kato, Diffusion coefficient of oxygen in propylene glycol-water mixtures and glycerol-water mixtures. Yakugaku Zasshi 82, 120–126 (1962). 14480206 10.1248/yakushi1947.82.1_120

[30] G. Vankó, T. Neisius, G. Molnár, F. Renz, S. Kárpáti, A. Shukla, F. M. F. de Groot, Probing the 3D spin momentum with X-ray emission spectroscopy: The case of molecular-spin transitions. J. Phys. Chem. B 110, 11647–11653 (2006). 16800459 10.1021/jp0615961

[31] V. Srinivas, R. Banerjee, H. Lebrette, J. C. Jones, O. Aurelius, I. S. Kim, C. C. Pham, S. Gul, K. D. Sutherlin, A. Bhowmick, J. John, E. Bozkurt, T. Fransson, P. Aller, A. Butryn, I. Bogacz, P. Simon, S. Keable, A. Britz, K. Tono, K. S. Kim, S. Y. Park, S. J. Lee, J. Park, R. Alonso-Mori, F. D. Fuller, A. Batyuk, A. S. Brewster, U. Bergmann, N. K. Sauter, A. M. Orville, V. K. Yachandra, J. Yano, J. D. Lipscomb, J. Kern, M. Högbom, High-resolution XFEL structure of the soluble methane monooxygenase hydroxylase complex with its regulatory component at ambient temperature in two oxidation states. J. Am. Chem. Soc. 142, 14249–14266 (2020). 32683863 10.1021/jacs.0c05613 PMC7457426

[32] S. Lafuerza, A. Carlantuono, M. Retegan, P. Glatzel, Chemical sensitivity of Kβ and Kα X-ray emission from a systematic investigation of iron compounds. Inorg. Chem. 59, 12518–12535 (2020). 32830953 10.1021/acs.inorgchem.0c01620

[33] L. A. McNeill, T. J. N. Brown, M. Sami, I. J. Clifton, N. I. Burzlaff, T. D. W. Claridge, R. M. Adlington, J. E. Baldwin, P. J. Rutledge, C. J. Schofield, Terminally truncated isopenicillin N synthase generates a dithioester product: Evidence for a thioaldehyde intermediate during catalysis and a new mode of reaction for non-heme iron oxidases. Chemistry 23, 12815–12824 (2017). 28703303 10.1002/chem.201701592 PMC5637899

[34] A. M. Rydzik, J. Brem, S. S. van Berkel, I. Pfeffer, A. Makena, T. D. W. Claridge, C. J. Schofield, Monitoring conformational changes in the NDM-1 Metallo-β-lactamase by ^19^F NMR spectroscopy. Angew. Chem. Int. Ed. 53, 3129–3133 (2014). 10.1002/anie.201310866 PMC4499255 24615874

[35] D. Roelofs, M. J. T. N. Timmermans, P. Hensbergen, H. van Leeuwen, J. Koopman, A. Faddeeva, W. Suring, T. E. de Boer, J. Mariën, R. Boer, R. Bovenberg, N. M. van Straalen, A functional isopenicillin N synthase in an animal genome. Mol. Biol. Evol. 30, 541–548 (2013). 23204388 10.1093/molbev/mss269

[36] J. P. Klinman, Dynamically achieved active site precision in enzyme catalysis. Acc. Chem. Res. 48, 449–456 (2015). 25539048 10.1021/ar5003347 PMC4334267

[37] J. E. Baldwin, J. M. Blackburn, J. D. Sutherland, M. C. Wright, High-level soluble expression of isopenicillin N synthase isozymes in *E. coli*. Tetrahedron 47, 5991–6002 (1991).

[38] I. Pettinati, P. Grzechnik, C. Ribeiro de Almeida, J. Brem, M. A. McDonough, S. Dhir, N. J. Proudfoot, C. J. Schofield, Biosynthesis of histone messenger RNA employs a specific 3′ end endonuclease. eLife 7, (2018). 10.7554/eLife.39865 PMC6303110 30507380

[39] M. R. Thomas, S. G. Boxer, 19F NMR of trifluoroacetyl-labeled cysteine mutants of myoglobin: Structural probes of nitric oxide bound to the H93G cavity mutant. Biochemistry 40, 8588–8596 (2001). 11456499 10.1021/bi0101087

[40] R. K. Lesniak, A. M. Rydzik, J. J. A. G. Kamps, A. Kahn, T. D. W. Claridge, C. J. Schofield, ^19^F NMR studies on γ-butyrobetaine hydroxylase provide mechanistic insights and suggest a dual inhibition mode. Chem. Commun. 55, 14717–14720 (2019). 10.1039/c9cc06466d PMC6927413 31702759

[41] E. van Groesen, C. T. Lohans, J. Brem, K. M. J. Aertker, T. D. W. Claridge, C. J. Schofield, ^19^F NMR monitoring of reversible protein post-translational modifications: Class D β-lactamase carbamylation and inhibition. Chemistry 25, 11837–11841 (2019). 31310409 10.1002/chem.201902529 PMC6771976

[42] S. J. Walmsley, P. A. Rudnick, Y. Liang, Q. Dong, S. E. Stein, A. I. Nesvizhskii, Comprehensive analysis of protein digestion using six trypsins reveals the origin of trypsin as a significant source of variability in proteomics. J. Proteome Res. 12, 5666–5680 (2013). 24116745 10.1021/pr400611h PMC4076643

[43] J. Gault, J. A. C. Donlan, I. Liko, J. T. S. Hopper, K. Gupta, N. G. Housden, W. B. Struwe, M. T. Marty, T. Mize, C. Bechara, Y. Zhu, B. Wu, C. Kleanthous, M. Belov, E. Damoc, A. Makarov, C. V. Robinson, High-resolution mass spectrometry of small molecules bound to membrane proteins. Nat. Methods 13, 333–336 (2016). 26901650 10.1038/nmeth.3771 PMC4856209

[44] G. Winter, C. M. C. Lobley, S. M. Prince, Decision making in xia2. Acta Crystallogr D 69, 1260–1273 (2013). 23793152 10.1107/S0907444913015308 PMC3689529

[45] A. Dubus, M. Sami, T. J. N. Brown, C. J. Schofield, J. E. Baldwin, J. M. Frère, Studies of isopenicillin N synthase enzymatic properties using a continuous spectrophotometric assay. FEBS Lett. 485, 142–146 (2000). 11094156 10.1016/s0014-5793(00)02221-3

[46] P. Emma, R. Akre, J. Arthur, R. Bionta, C. Bostedt, J. Bozek, A. Brachmann, P. Bucksbaum, R. Coffee, F. J. Decker, Y. Ding, D. Dowell, S. Edstrom, A. Fisher, J. Frisch, S. Gilevich, J. Hastings, G. Hays, P. Hering, Z. Huang, R. Iverson, H. Loos, M. Messerschmidt, A. Miahnahri, S. Moeller, H. D. Nuhn, G. Pile, D. Ratner, J. Rzepiela, D. Schultz, T. Smith, P. Stefan, H. Tompkins, J. Turner, J. Welch, W. White, J. Wu, G. Yocky, J. Galayda, First lasing and operation of an Ångstrom-wavelength free-electron laser. Nat. Photonics 4, 641–647 (2010).

[47] S. Boutet, A. Cohen, S. Wakatsuki, The new macromolecular femtosecond crystallography (mfx) instrument at LCLS. Synchrotron. Radiat. News 29, 23–28 (2016). 28736484 10.1080/08940886.2016.1124681 PMC5519296

[48] M. Sugahara, E. Mizohata, E. Nango, M. Suzuki, T. Tanaka, T. Masuda, R. Tanaka, T. Shimamura, Y. Tanaka, C. Suno, K. Ihara, D. Pan, K. Kakinouchi, S. Sugiyama, M. Murata, T. Inoue, K. Tono, C. Song, J. Park, T. Kameshima, T. Hatsui, Y. Joti, M. Yabashi, S. Iwata, Grease matrix as a versatile carrier of proteins for serial crystallography. Nat. Methods 12, 61–63 (2015). 25384243 10.1038/nmeth.3172

[49] A. S. Brewster, I. D. Young, A. Lyubimov, A. Bhowmick, N. K. Sauter, Processing serial crystallographic data from XFELs or synchrotrons using the *cctbx.xfel* GUI. Comput. Crystallogr. News. 10, 22–39 (2019).

[50] N. K. Sauter, J. Hattne, R. W. Grosse-Kunstleve, N. Echols, New Python-based methods for data processing. Acta Crystallogr. D Biol. Crystallogr. 69, 1274–1282 (2013). 23793153 10.1107/S0907444913000863 PMC3689530

[51] J. Hattne, N. Echols, R. Tran, J. Kern, R. J. Gildea, A. S. Brewster, R. Alonso-Mori, C. Glöckner, J. Hellmich, H. Laksmono, R. G. Sierra, B. Lassalle-Kaiser, A. Lampe, G. Han, S. Gul, D. DiFiore, D. Milathianaki, A. R. Fry, A. Miahnahri, W. E. White, D. W. Schafer, M. M. Seibert, J. E. Koglin, D. Sokaras, T. C. Weng, J. Sellberg, M. J. Latimer, P. Glatzel, P. H. Zwart, R. W. Grosse-Kunstleve, M. J. Bogan, M. Messerschmidt, G. J. Williams, S. Boutet, J. Messinger, A. Zouni, J. Yano, U. Bergmann, V. K. Yachandra, P. D. Adams, N. K. Sauter, Accurate macromolecular structures using minimal measurements from X-ray free-electron lasers. Nat. Methods 11, 545–548 (2014). 24633409 10.1038/nmeth.2887 PMC4008696

[52] N. K. Sauter, XFEL diffraction: Developing processing methods to optimize data quality. J. Synchrotron Radiat. 22, 239–248 (2015). 25723925 10.1107/S1600577514028203 PMC4344359

[53] G. Winter, D. G. Waterman, J. M. Parkhurst, A. S. Brewster, R. J. Gildea, M. Gerstel, L. Fuentes-Montero, M. Vollmar, T. Michels-Clark, I. D. Young, N. K. Sauter, G. Evans, DIALS: Implementation and evaluation of a new integration package. Acta Crystallogr. D Struct. Biol. 74, 85–97 (2018). 29533234 10.1107/S2059798317017235 PMC5947772

[54] N. K. Sauter, J. Hattne, A. S. Brewster, N. Echols, P. H. Zwart, P. D. Adams, Improved crystal orientation and physical properties from single-shot XFEL stills. Acta Crystallogr. D 70, 3299–3309 (2014). 25478847 10.1107/S1399004714024134 PMC4257623

[55] D. G. Waterman, G. Winter, R. J. Gildea, J. M. Parkhurst, A. S. Brewster, N. K. Sauter, G. Evans, Diffraction-geometry refinement in the *DIALS* framework. Acta Crystallogr. D Struct. Biol. 72, 558–575 (2016). 27050135 10.1107/S2059798316002187 PMC4822564

[56] A. S. Brewster, D. G. Waterman, J. M. Parkhurst, R. J. Gildea, I. D. Young, L. J. O’Riordan, J. Yano, G. Winter, G. Evans, N. K. Sauter, Improving signal strength in serial crystallography with *DIALS* geometry refinement. Acta Crystallogr. D Struct. Biol. 74, 877–894 (2018). 30198898 10.1107/S2059798318009191 PMC6130462

[57] M. Uervirojnangkoorn, O. B. Zeldin, A. Y. Lyubimov, J. Hattne, A. S. Brewster, N. K. Sauter, A. T. Brunger, W. I. Weis, Enabling X-ray free electron laser crystallography for challenging biological systems from a limited number of crystals. eLife 4, e05421 (2015). 10.7554/eLife.05421 PMC4397907 25781634

[58] A. J. McCoy, R. W. Grosse-Kunstleve, P. D. Adams, M. D. Winn, L. C. Storoni, R. J. Read, Phaser crystallographic software. J. Appl. Cryst. 40, 658–674 (2007). 19461840 10.1107/S0021889807021206 PMC2483472

[59] P. D. Adams, R. W. Grosse-Kunstleve, L. W. Hung, T. R. Ioerger, A. J. McCoy, N. W. Moriarty, R. J. Read, J. C. Sacchettini, N. K. Sauter, T. C. Terwilliger, PHENIX: Building new software for automated crystallographic structure determination. Acta Crystallogr. D Biol. Crystallogr. 58, 1948–1954 (2002). 12393927 10.1107/s0907444902016657

[60] P. Emsley, B. Lohkamp, W. G. Scott, K. Cowtan, Features and development of Coot. Acta Crystallogr. D Biol. Crystallogr. 66, 486–501 (2010). 20383002 10.1107/S0907444910007493 PMC2852313

[61] R. H. B. Smith, A. C. Dar, A. Schlessinger, PyVOL: A PyMOL plugin for visualization, comparison, and volume calculation of drug-binding sites. bioRxiv 2019, 816702 (2019).

[62] R. L. Owen, D. Axford, D. A. Sherrell, A. Kuo, O. P. Ernst, E. C. Schulz, R. J. D. Miller, H. M. Mueller-Werkmeister, Low-dose fixed-target serial synchrotron crystallography. Acta Crystallogr. D Struct. Biol. 73, 373–378 (2017). 28375148 10.1107/S2059798317002996 PMC5379936

[63] D. Liebschner, P. V. Afonine, N. W. Moriarty, B. K. Poon, O. V. Sobolev, T. C. Terwilliger, P. D. Adams, Polder maps: Improving OMIT maps by excluding bulk solvent. Acta Crystallogr. D Struct. Biol. 73, 148–157 (2017). 28177311 10.1107/S2059798316018210 PMC5297918

[64] C. R. Randall, Y. Zang, A. E. True, L. Que Jr., J. M. Charnock, C. D. Garner, Y. Fujishima, C. J. Schofield, J. E. Baldwin, X-ray absorption studies of the ferrous active site of isopenicillin N synthase and related model complexes. Biochemistry 32, 6664–6673 (1993). 8329393 10.1021/bi00077a020

[65] A. M. Orville, V. J. Chen, A. Kriauciunas, M. R. Harpel, B. G. Fox, E. Munck, J. D. Lipscomb, Thiolate ligation of the active site iron(II) of isopenicillin N synthase derives from substrate rather than endogenous cysteine: Spectroscopic studies of site-specific Cys → Ser mutated enzymes. Biochemistry 31, 4602–4612 (1992). 1316153 10.1021/bi00134a010

[66] S. F. Altschul, W. Gish, W. Miller, E. W. Myers, D. J. Lipman, Basic local alignment search tool. J. Mol. Biol. 215, 403–410 (1990). 2231712 10.1016/S0022-2836(05)80360-2

[67] P. Gouet, X. Robert, E. Courcelle, ESPript/ENDscript: Extracting and rendering sequence and 3D information from atomic structures of proteins. Nucleic Acids Res. 31, 3320–3323 (2003). 12824317 10.1093/nar/gkg556 PMC168963

[68] K. Valegard, A. C. van Scheltinga, M. D. Lloyd, T. Hara, S. Ramaswamy, A. Perrakis, A. Thompson, H. J. Lee, J. E. Baldwin, C. J. Schofield, J. Hajdu, I. Andersson, Structure of a cephalosporin synthase. Nature 394, 805–809 (1998). 9723623 10.1038/29575

[69] C. G. Yang, C. Yi, E. M. Duguid, C. T. Sullivan, X. Jian, P. A. Rice, C. He, Crystal structures of DNA/RNA repair enzymes AlkB and ABH2 bound to dsDNA. Nature 452, 961–965 (2008). 18432238 10.1038/nature06889 PMC2587245

[70] R. Chowdhury, M. A. McDonough, J. Mecinović, C. Loenarz, E. Flashman, K. S. Hewitson, C. Domene, C. J. Schofield, Structural basis for binding of hypoxia-inducible factor to the oxygen-sensing prolyl hydroxylases. Structure 17, 981–989 (2009). 19604478 10.1016/j.str.2009.06.002

[71] J. R. Horton, A. K. Upadhyay, H. H. Qi, X. Zhang, Y. Shi, X. Cheng, Enzymatic and structural insights for substrate specificity of a family of jumonji histone lysine demethylases. Nat. Struct. Mol. Biol. 17, 38–43 (2010). 20023638 10.1038/nsmb.1753 PMC2849977

[72] H. Hashimoto, J. E. Pais, X. Zhang, L. Saleh, Z. Q. Fu, N. Dai, I. R. Corrêa, Y. Zheng, X. Cheng, Structure of a Naegleria Tet-like dioxygenase in complex with 5-methylcytosine DNA. Nature 506, 391–395 (2014). 24390346 10.1038/nature12905 PMC4364404

[73] M. A. McDonough, K. L. Kavanagh, D. Butler, T. Searls, U. Oppermann, C. J. Schofield, Structure of human phytanoyl-CoA 2-hydroxylase identifies molecular mechanisms of Refsum disease. J. Biol. Chem. 280, 41101–41110 (2005). 16186124 10.1074/jbc.M507528200

[74] D. H. Juers, J. Ruffin, MAP_CHANNELS: A computation tool to aid in the visualization and characterization of solvent channels in macromolecular crystals. J. Appl. Cryst. 47, 2105–2108 (2014). 25484846 10.1107/S160057671402281X PMC4248570

